# Proteasome-Dependent Disruption of the E3 Ubiquitin Ligase Anaphase-Promoting Complex by HCMV Protein pUL21a

**DOI:** 10.1371/journal.ppat.1002789

**Published:** 2012-07-05

**Authors:** Anthony R. Fehr, Nathaniel C. Gualberto, John Paul Savaryn, Scott S. Terhune, Dong Yu

**Affiliations:** 1 Department of Molecular Microbiology, Washington University School of Medicine, St. Louis, Missouri, United States of America; 2 Department of Microbiology and Molecular Genetics, Medical College of Wisconsin, Milwaukee, Wisconsin, United States of America; 3 Biotechnology and Bioengineering Center, Medical College of Wisconsin, Milwaukee, Wisconsin, United States of America; McMaster University, Canada

## Abstract

The anaphase-promoting complex (APC) is an E3 ubiquitin ligase which controls ubiquitination and degradation of multiple cell cycle regulatory proteins. During infection, human cytomegalovirus (HCMV), a widespread pathogen, not only phosphorylates the APC coactivator Cdh1 via the multifunctional viral kinase pUL97, it also promotes degradation of APC subunits via an unknown mechanism. Using a proteomics approach, we found that a recently identified HCMV protein, pUL21a, interacted with the APC. Importantly, we determined that expression of pUL21a was necessary and sufficient for proteasome-dependent degradation of APC subunits APC4 and APC5. This resulted in APC disruption and required pUL21a binding to the APC. We have identified the proline-arginine amino acid pair at residues 109–110 in pUL21a to be critical for its ability to bind and regulate the APC. A point mutant virus in which proline-arginine were mutated to alanines (PR-AA) grew at wild-type levels. However, a double mutant virus in which the viral ability to regulate the APC was abrogated by both PR-AA point mutation and UL97 deletion was markedly more attenuated compared to the UL97 deletion virus alone. This suggests that these mutations are synthetically lethal, and that HCMV exploits two viral factors to ensure successful disruption of the APC to overcome its restriction on virus infection. This study reveals the HCMV protein pUL21a as a novel APC regulator and uncovers a unique viral mechanism to subvert APC activity.

## Author Summary

In this study, we report an intriguing mechanism used by human cytomegalovirus (HCMV) to regulate a cellular E3 ubiquitin ligase, the anaphase promoting complex (APC). The ability to hijack the ubiquitin-proteasome system for regulating protein degradation and to manipulate the cell cycle for viral genome synthesis is critical in many viral infections. The APC is a master cell cycle modulator that targets a number of regulatory proteins for proteasomal degradation. It can prevent cells from entry into S-phase, thus creating a hindrance for viruses needing to coerce cells into a cellular environment favorable for viral DNA synthesis. We have identified an HCMV protein, pUL21a, which uses a seemingly counterintuitive mechanism to regulate the APC. It interacts with the APC to target the subunits of this ubiquitin ligase for proteasomal degradation. This causes disruption of the complex and reduces its activity. Furthermore, a virus lacking pUL21a and pUL97, which is another HCMV-encoded APC regulator, was highly attenuated when compared to loss of UL97 alone, suggesting that HCMV uses two proteins to fully disarm the APC. This study identifies a herpesviral protein that uses a unique, proteasome-dependent mechanism to regulate the activity of this prominent cellular E3 ubiquitin ligase.

## Introduction

Regulation of protein degradation plays a key role in many cellular processes ranging from cell cycle progression, innate immunity, and antigen presentation to the turnover of misfolded or oxidized proteins. Most degradation is carried out by the ubiquitin-proteasome system (UPS). Ubiquitin is added to proteins by a cascade of ubiquitin conjugating enzymes, resulting in a polyubiquitinated protein which is subsequently degraded by the 26S proteasome. As a means to regulate protein function, it is no surprise that many viruses have co-opted the UPS for their own benefit. Viruses can promote proteasome degradation of antiviral host proteins either by encoding their own E3 ubiquitin ligase, targeting proteins to a cellular E3 ligase, or even inducing ubiquitin-independent degradation of targets. Examples of viral E3 ligases include the herpes simplex virus-1 protein ICP0 [1] and Kaposi's sarcoma-associated herpesvirus proteins K3 and K5 (for a review, see [2]). Viral proteins that can hijack a cellular E3 ligase include human immunodeficiency virus-1 vpr and vif (for a review, see [3]), paramyxovirus V [4], and human papillomavirus E6 and E7 (for a review, see [5]). Finally, the human cytomegalovirus (HCMV) protein pp71 uses a ubiquitin-independent mechanism to target the Rb and hDaxx proteins [6], [7]. In fact, pharmacological inhibition of the proteasome blocks multiple stages of the viral life cycle, suggesting that viruses rely on activities of the UPS for their replication [8]–[12]. On the other hand, viruses must also modulate cellular E3 ligase activity in order to replicate because ubiquitination regulates many important cellular processes central to virus infection. The SV40 large T antigen inhibits the SCF^fbw7^ ubiquitin ligase to increase cyclin E levels [13], and influenza virus NS1 inhibits TRIM 25-mediated ubiquitination of RIG-I, thereby attenuating interferon production [14].

The anaphase-promoting complex (APC) or cyclosome is a macromolecular complex that contains cullin-ring E3 ubiquitin ligase activity and is conserved across all eukaryotes (for a review, see [15]). It has at least eleven subunits and two co-activator proteins (CDC20 (cell-division cycle protein 20) and Cdh1 (CDC20 homologue 1)), which are separated into three sub-complexes. These include the cullin-ring ligase domain (composed of APC2, 10, and 11), the specificity arm (composed of APC3, 6, 7, and 8), and the bridge (composed of APC1, 4, and 5). Cdh1 and CDC20 activate APC activity to prevent premature entry into S phase and to promote progression through mitosis, respectively. The APC complex ubiquitinates more than 40 proteins, including A- and B- type cyclins, to regulate their stability. It also regulates degradation of its own coactivator proteins, Cdh1 and CDC20, as a form of feedback regulation. Due to its central role in cell cycle progression, the APC is also a promising target for anti-cancer therapeutics [16].

Many viruses modulate the host cell cycle to establish optimal conditions for their replication. Several viral proteins have been reported to target the APC, possibly to force the cell into an S phase-like biochemical environment to promote efficient viral replication. Proteins from adenovirus, chicken anemia virus, human papillomavirus, human T-lymphotropic virus, hepatitis B virus, parapoxvirus, and HCMV have been reported to regulate the function of the APC [17]–[24]. However, the mechanisms used by these viruses during infection to subvert the APC are largely unknown.

HCMV is a globally important opportunistic pathogen that causes severe diseases in immunocompromised individuals and is the leading viral cause of congenital diseases. This virus stimulates cell cycle progression of quiescent cells into an S phase-like environment but concurrently blocks host DNA synthesis [25]. HCMV promotes cell cycle progression likely in part by inactivating Rb [6], [26] and regulating the APC [24], [27], [28]. It has been reported that HCMV has two means to regulate the APC. The multifunctional viral kinase pUL97 phosphorylates the APC coactivator Cdh1, thus likely inhibiting its activity [24]. Nonetheless, abrogation of UL97 alone only results in a modest increase in APC activity during infection [24]. Independent of UL97-mediated Cdh1 regulation, HCMV also induces degradation of two APC subunits, APC4 and APC5, leading to the dissociation of the complex during infection [24]. The viral factor and associated mechanism responsible for regulating degradation of the APC subunits have not been identified.

In this study, we demonstrate that the HCMV protein pUL21a interacts with the APC, resulting in proteasome-dependent degradation of APC4 and APC5. Expression of pUL21a dissociates the APC cullin-ring ligase subcomplex from its specificity arm. This regulation alters APC activity and increases levels of a subset of APC-regulated cell cycle proteins. We have identified residues proline-arginine (PR109-110) in pUL21a to be critical for its ability to bind and regulate the APC. A mutant virus in which the viral ability to regulate the APC is abrogated by both alanine substitution of proline-arginine residues in pUL21a and UL97 deletion is markedly more defective compared to the pUL97 deletion virus alone. This suggests that HCMV has evolved an invasive strategy of using both viral factors to regulate the APC to facilitate its infection. Our study has identified the HCMV protein pUL21a as a novel APC regulator and elucidated a unique mechanism to subvert APC activity.

## Results

### pUL21a Interacts with the Anaphase-Promoting Complex (APC)

**Figure 1:**
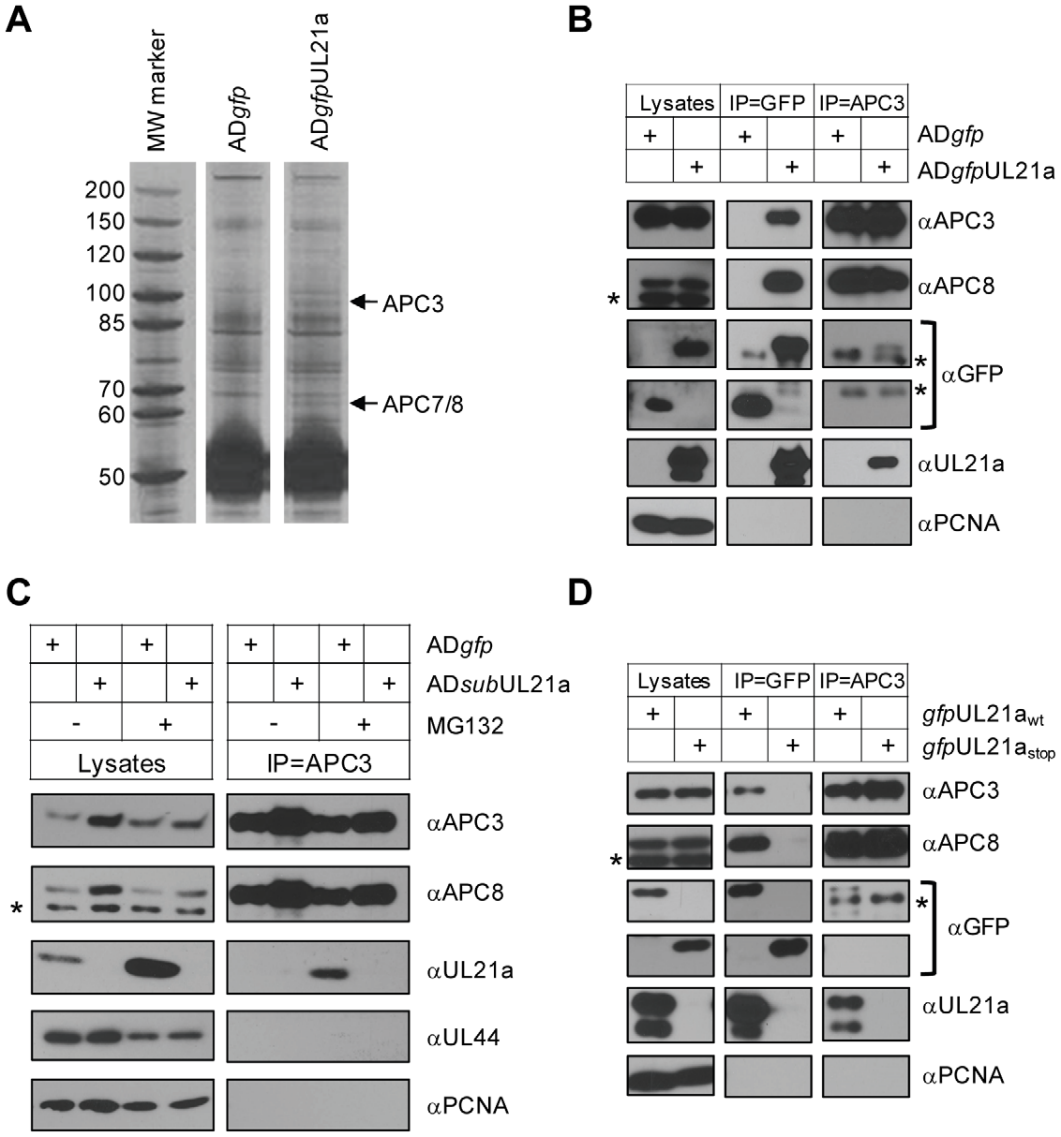
pUL21a interacts with the APC. (A) Identification of pUL21a interacting partners. MRC-5 cells were infected with AD*gfp* or AD*gfp*UL21a at an MOI of 5, collected at 48 hpi, and were immunoprecipitated with GFP antibody. Eluted proteins were run on an SDS-containing polyacrylamide gel and silver stained. The bands indicated with an arrow were identified by mass spectrometry as APC3 (100 kDa), APC7, and APC8 (both at 65 kDa). (B) GFP-tagged pUL21a interacts with the APC in HCMV infection. MRC-5 cells were infected as described in panel A, and lysates were subjected to co-immunoprecipitation with GFP or APC3 mouse monoclonal antibodies. Cell lysates and eluted proteins were analyzed by immunoblotting with indicated antibodies. GFP blots were cropped to save space but were from the same lane and exposed film. Non-specific cross-reacting bands are indicated by asterisk (see text). Partial proteolysis was often seen with the GFP-tagged UL21a protein, particularly in cell lysate samples. (C) Native pUL21a interacts with the APC in HCMV infection. MRC-5 cells were infected with AD*gfp* or AD*sub*UL21a (as described in Materials and Methods) in the presence (+) or absence (−) of 10 µM MG132. Cell lysates were prepared at 24 hpi and immunoprecipitated with APC3 antibody. Cell lysates and eluted proteins were analyzed by immunoblotting. (D) Interaction of pUL21a with the APC does not require other viral proteins. 293T cells were transfected with plasmids expressing *gfp*UL21a_wt_ or *gfp*UL21a_stop_. Cells were collected 72 hours later and cell lysates were immunoprecipitated as in panel B. Cell lysates and eluted proteins were analyzed by immunoblotting. PCNA and pUL44 were used as cellular and viral negative controls, respectively.

HCMV pUL21a is a 15 kDa, highly unstable protein that is expressed with early kinetics [29]. One identified function of this protein is to facilitate efficient viral DNA synthesis [30]. However, this protein shares no significant homology with any known protein. To provide mechanistic insight into its activity, we used a proteomics approach to identify interacting partners of pUL21a during infection. We created a recombinant virus (AD*gfp*UL21a) in which the UL21a coding sequence was tagged with the green fluorescent protein (GFP) coding sequence. This virus grew with wild-type kinetics, and the tagged protein was fortuitously much more stable than native pUL21a [29]. A GFP tag can stabilize certain fusion proteins [31], and made it possible to detect interacting proteins in our study. We infected fibroblasts with either AD*gfp*UL21a or control HCMV (AD*gfp*) that expressed free GFP only. At 48 hours post infection (hpi), we isolated the protein complexes from infected cells by a rapid one-step immunoaffinity purification on magnetic beads coated with GFP antibody-coupled protein A. Electrophoresis analysis revealed multiple protein bands that were specific to the pUL21a-containing sample (Figure 1A). We analyzed pUL21a-specific protein bands by mass spectrometry and identified the proteins depicted with arrows as APC specificity arm subunits, APC3, APC7, and APC8 (Table S1).

We validated these interactions in HCMV infected cells by co-immunoprecipitation followed by immunoblot analysis. Here we used APC3 and APC8 as the marker for the APC complex. Pull-down of pGFP-UL21a, but not the GFP control, isolated both APC3 and APC8 (Figure 1B). The lower band detected by APC8 antibody was nonspecific as it neither co-immunoprecipitated with APC3 antibody (Figure 1B) nor was affected by shRNA knockdown of APC8 (Figure S1). In the reciprocal experiment, APC3 antibody co-immunoprecipitated APC8 and pGFP-UL21a but not GFP. Neither GFP nor APC3 antibody co-immunoprecipitated cellular PCNA (Figure 1B), and an antibody against HA did not co-immunoprecipitate any of the proteins detected here (data not shown), thus providing additional evidence for the specificity of these interactions. As pGFP-UL21a is co-immunoprecipitated with multiple APC subunits, we interpret the result to suggest that pUL21a binds to the APC complex, even though the precise subunit where pUL21a directly interacts with remains unknown.

To determine if this interaction also occurred with native pUL21a, we performed co-immunoprecipitation assays on lysates from cells infected with wild-type virus (AD*gfp*), and we also included lysate from cells infected with UL21a deletion virus (AD*sub*UL21a) as a negative control (Figure 1C). Infected cells were treated with proteasome inhibitor, MG132, as pUL21a was highly unstable and otherwise could not accumulate to levels allowing reproducible detection of this interaction [29]. In the presence of MG132, the level of native pUL21a was markedly increased and could be co-immunoprecipitated with APC3 antibody. This interaction was specific as the antibody did not co-immunoprecipitate PCNA or the viral DNA polymerase accessory factor UL44.

To test if pUL21a was able to bind to the APC in the absence of other HCMV proteins, we performed co-immunoprecipitation assay on lysates from 293T cells transfected with constructs expressing the GFP-amino terminal tagged UL21a (*gfp*UL21a_wt_) or UL21a carrying two stop codons at its amino terminus to abrogate pUL21a expression (*gfp*UL21a_stop_) (Figure 1D). Both *gfp*UL21a_wt_ and *gfp*UL21a_stop_ were expressed at equal levels but only *gfp*UL21a_wt_ associated with APC3 or APC8. Additionally, APC3 antibody co-immunoprecipitated *gfp*UL21a_wt_ but not *gfp*UL21a_stop_. We conclude that pUL21a interacts with the APC and does not require other HCMV proteins for this interaction to occur.

### The Carboxyl-Terminus of pUL21a Contains the APC Binding Site

**Figure 2:**
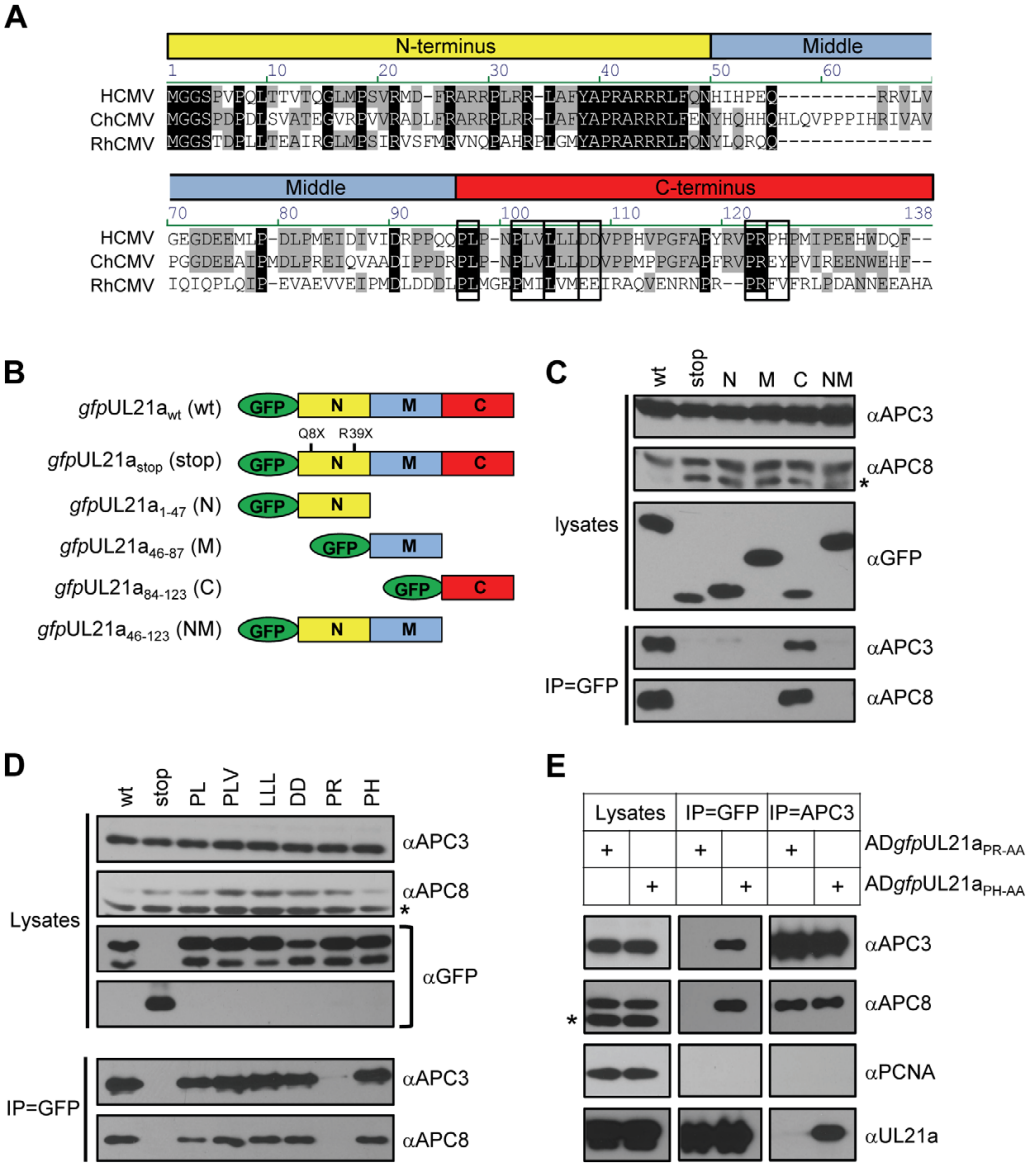
The carboxyl-terminus of pUL21a contains the APC binding site. (A) Amino acid alignment of UL21a proteins from human, chimpanzee, and rhesus CMVs. Boxes above aligned proteins divide the protein into N-terminal, Middle, and C-terminal regions. Conserved residues at the C-terminal region targeted for alanine substitution are boxed. (B) Diagram of UL21a truncation mutants analyzed in this study. (C) The C-terminus of pUL21a binds to the APC. GFP-tagged UL21a truncation mutant proteins were expressed in 293T cells by transfection, cells were collected at 72 hours, and lysates were immunoprecipitated with GFP antibody. Cell lysates and eluted proteins were analyzed by immunoblotting. (D) Identification of residues critical for APC binding. C-terminal conserved residues indicated in panel A were mutated by alanine substitution, and GFP-tagged UL21a mutant proteins were tested for APC binding as described in panel C. (E) The APC binding site of pUL21a was validated during HCMV infection. MRC-5 cells were infected with recombinant HCMV virus carrying the GFP-tagged UL21a_PH-AA_ or UL21a_PR-AA_ point mutant. Cells were collected at 48 hpi and lysates were immunoprecipitated with GFP or APC3 antibody. Cell lysates and eluted proteins were analyzed by immunoblotting. Non-specific cross-reacting bands are indicated by asterisk.

To begin understanding the nature of this interaction, we identified the APC-binding domain of pUL21a. Sequence alignment of pUL21a with its homologues in chimpanzee CMV (CCMV) and Rhesus CMV (RhCMV) revealed a highly conserved N-terminus (residues 1–47), divergent middle region (residues 48–83), and C-terminus that contained several conserved residues (residues 84–123), including a proline-arginine (PR) pair at residues 109–110 (Figure 2A). We created a series of truncation mutations targeting each region in the GFP-tagged pUL21a, and tested the ability of mutant UL21a proteins to interact with the APC in 293T cells (Figure 2B). All mutants were expressed at similar levels and were efficiently immunoprecipitated by the GFP antibody (Figure 2C, and data not shown). As expected, full-length *gfp*UL21a_wt_ co-immunoprecipitated both APC3 and APC8 while the *gfp*UL21a_stop_ mutant did not. Importantly, while the carboxyl-terminal fragment of pUL21a consistently co-immunoprecipitated APC3 and APC8, the amino-terminal and middle fragments were unable to do so. Thus the carboxyl-terminus of pUL21a contains the APC binding domain.

To define the precise sequence of the APC binding site, we made *gfp*UL21a mutants in which each of five conserved residue clusters within its carboxyl terminus were individually substituted with alanine residues (Figure 2A). As a control, we also made alanine substitutions for the non-conserved proline-histidine pair at residues 111–112 (PH111-112AA) (Figure 2A). All mutants were stable and were efficiently pulled down by the GFP antibody (Figure 2D, and data not shown). Among them, only the PR109-110AA mutant lost the ability to bind to the APC. Substitutions of the adjoining non-conserved residues (PH111-112AA) had no effect on APC binding. To validate the result in the context of infection, we constructed recombinant HCMV viruses expressing GFP-tagged or native forms of PR109-110AA or PH111-112AA pUL21a variants (AD*gfp*UL21a_PR-AA_, AD*gfp*UL21a_PH-AA_, AD*pm*UL21a_PR-AA_, and AD*pm*UL21a_PH-AA_). During infection, a reciprocal interaction between *gfp*UL21a_PH-AA_ and APC3 could be detected while *gfp*UL21a_PR-AA_ and APC3 did not interact (Figure 2E). Furthermore, untagged pUL21a_PH-AA_, but not pUL21a_PR-AA_, was co-immunoprecipitated with APC3 when stabilized by MG132 (Figure S2). Together, these results indicate that the carboxyl terminus of pUL21a contains the APC binding domain and the residues PR_109–110_ are critical for this binding.

### Binding of pUL21a to the APC Promotes Degradation of APC4 and APC5 Subunits

**Figure 3:**
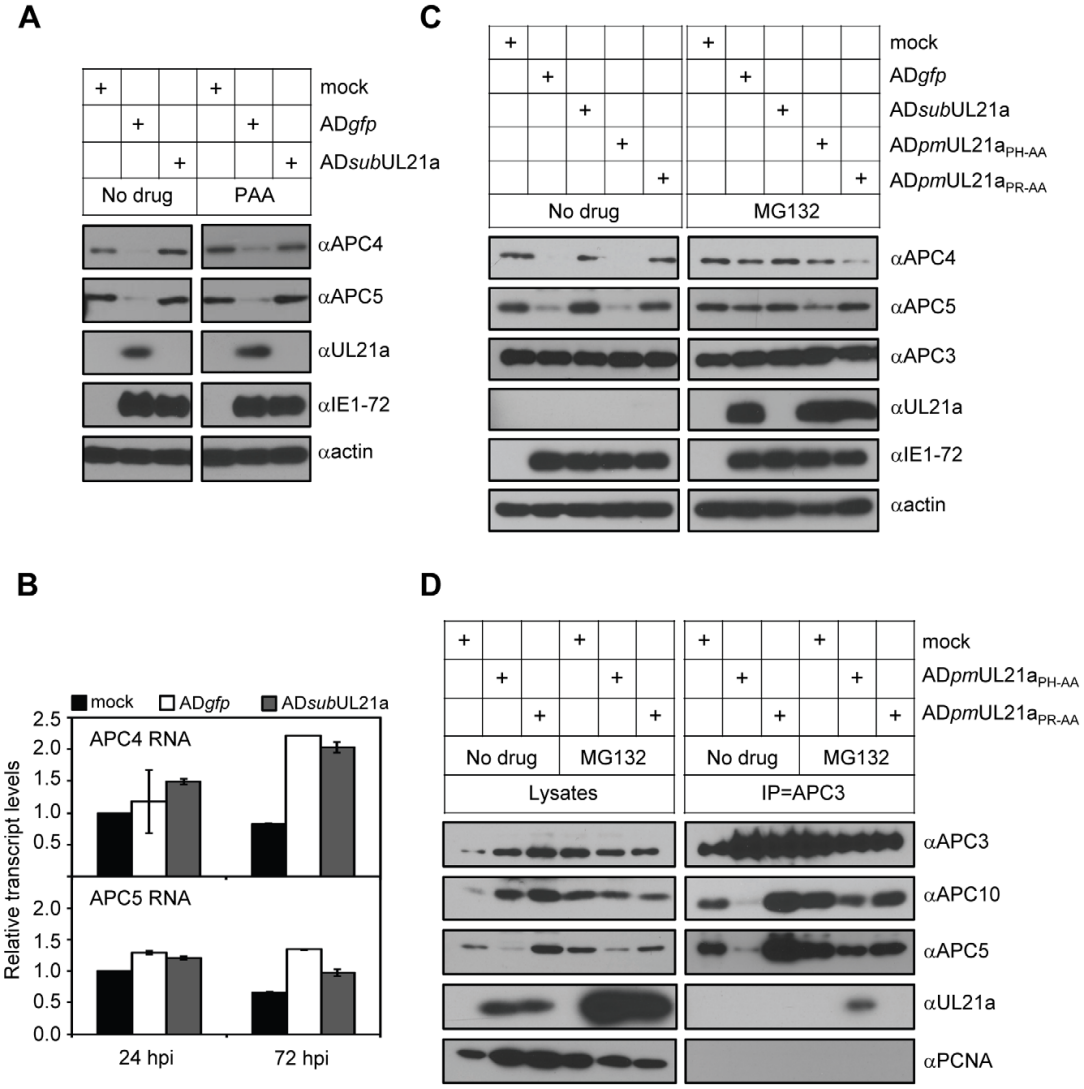
pUL21a binding to the APC promotes degradation of APC subunits and APC dissociation. (A) pUL21a is required for HCMV to reduce APC4 and APC5 accumulation during infection. MRC-5 cells were infected in the presence or absence of PAA with AD*gfp* or AD*sub*UL21a. Cell lysates were analyzed by immunoblotting at 24 hpi. pUL21a, IE1-72, and actin were used as infection and loading controls, respectively. (B) HCMV infection does not alter APC4 or APC5 transcript levels. MRC-5 cells were infected with AD*gfp* or AD*sub*UL21a, total RNA was collected at indicated times, and APC4 and APC5 transcripts were measured by reverse transcription-coupled quantitative PCR (RT-qPCR) and normalized to that of GAPDH. (C) APC binding activity of pUL21a is required for proteasome-dependent degradation of APC4 and APC5 in HCMV infection. MRC-5 cells were infected with AD*gfp*, AD*sub*UL21a, AD*pm*UL21a_PH-AA_, or AD*pm*UL21a_PR-AA_. MG132 was added at 6 hpi, and cell lysates were analyzed at 20 hpi by immunoblotting. (D) APC binding ability of pUL21a is required for APC dissociation in HCMV infection. MRC-5 cells were infected with AD*pm*UL21a_PH-AA_ or AD*pm*UL21a_PR-AA_, and treated with MG132 as described in panel C. Cells were collected at 20 hpi, cell lysates were immunoprecipitated with APC3 antibody, and both lysates and eluted proteins were analyzed by immunoblotting.

It has recently been reported that the APC bridge subunits APC4 and APC5 are degraded during HCMV infection and the complex dissociates [24]. To test if pUL21a was required for these events, we first examined APC subunit accumulation during infection with or without pUL21a. Levels of APC4 and APC5 proteins were markedly reduced during wild-type infection relative to mock-infected cells at 24 hpi (Figure 3A). However, no reduction was observed in APC4 and APC5 levels during infection with the UL21a-deletion virus. The pUL21a-deficient virus fails to express late viral genes due to a defect in viral DNA synthesis [30]. To rule out any role of late genes in APC4 and APC5 degradation, we treated infected cells with phosphonoacetic acid (PAA) to block viral DNA synthesis and late gene expression. APC4 and APC5 levels were reduced during infection with wild-type virus but remained elevated during infection with the UL21a-deletion virus, even following PAA treatment. Furthermore, there was no appreciable difference in APC4 and APC5 transcript levels between wild-type and deletion virus infections (Figure 3B). These data suggest that the changes in APC4 and APC5 protein levels occur at the level of protein stability. Consistent with this hypothesis, MG132 enhanced APC4 and APC5 protein levels during infection with wild-type but not deletion virus (Figure 3C). Thus, pUL21a-mediated loss of APC4 and APC5 was due to proteasomal degradation. Moreover, the APC binding mutant virus AD*pm*UL21a_PR-AA_ was unable to degrade APC4 and APC5 while the AD*pm*UL21a_PH-AA_ virus was as efficient as the wild-type control virus. These data support the conclusion that pUL21a binding to the APC promotes proteasomal degradation of APC4 and APC5.

We next tested if the APC binding ability of pUL21a was also required for APC dissociation during infection. In this experiment, we used APC3 and APC10 as the marker for the specificity arm and cullin-ring ligase subcomplex of the APC, respectively. These two subcomplexes sit on opposite sides of the APC. APC10 has been proposed to bind APC substrates along with coactivator proteins, including Cdh1 [32]. APC10 associates with APC2 and APC11 of the ligase subcomplex, but its location in the inner cavity of the APC allows for contact with APC3 and APC6 of the specificity arm. In cells infected with AD*pm*UL21a_PR-AA_, total levels of APC3 and APC10 were similar to those in cells infected with AD*pm*UL21a_PH-AA_, allowing for a direct analysis of the efficiency of their association with the complex (Figure 3D). APC3 could not co-immunoprecipitate APC10 in AD*pm*UL21a_PH-AA_-infected cells, consistent with dissociation of the complex in the presence of functional pUL21a. In cells infected with AD*pm*UL21a_PR-AA_, APC3 was able to pull down APC10 efficiently, indicating that the two subcomplexes remained associated. Finally, the integrity of the APC during AD*pm*UL21a_PH-AA_ infection was largely restored upon addition of MG132, even though total protein levels were reduced likely due to MG132-induced cell death (Figure 3D, and data not shown). These data were recapitulated during infection of wild-type and UL21a deletion viruses (Figure S3). Our data provides strong evidence supporting the model that binding of pUL21a to the APC induces degradation of the APC bridge arm resulting in complex dissociation.

As APC8 was co-immunoprecipitated with pUL21a in our original screen, it raised the possibility that pUL21a might require APC8 to target APC4 and APC5. For instance, pUL21a might bind to APC8 to disrupt the structure of the APC leading to APC4 and APC5 degradation, or it might use APC8 as a docking site for recruiting protein degradation enzymes to target APC4 and APC5. To test this, we depleted APC8 in these cells by shRNA knockdown (Figure S4). Following shRNA depletion of APC8, the APC4 and APC5 levels remained reduced in cells infected with wild-type virus compared to those with UL21a-deletion virus, even though APC knockdown did seem to affect the overall stability of APC4 and APC5 in pUL21a-independent manner (Figure S4). This suggests that pUL21a-mediated degradation of APC4 and APC5 is independent of APC8.

### pUL21a Expression Regulates APC Activity during HCMV Infection

**Figure 4:**
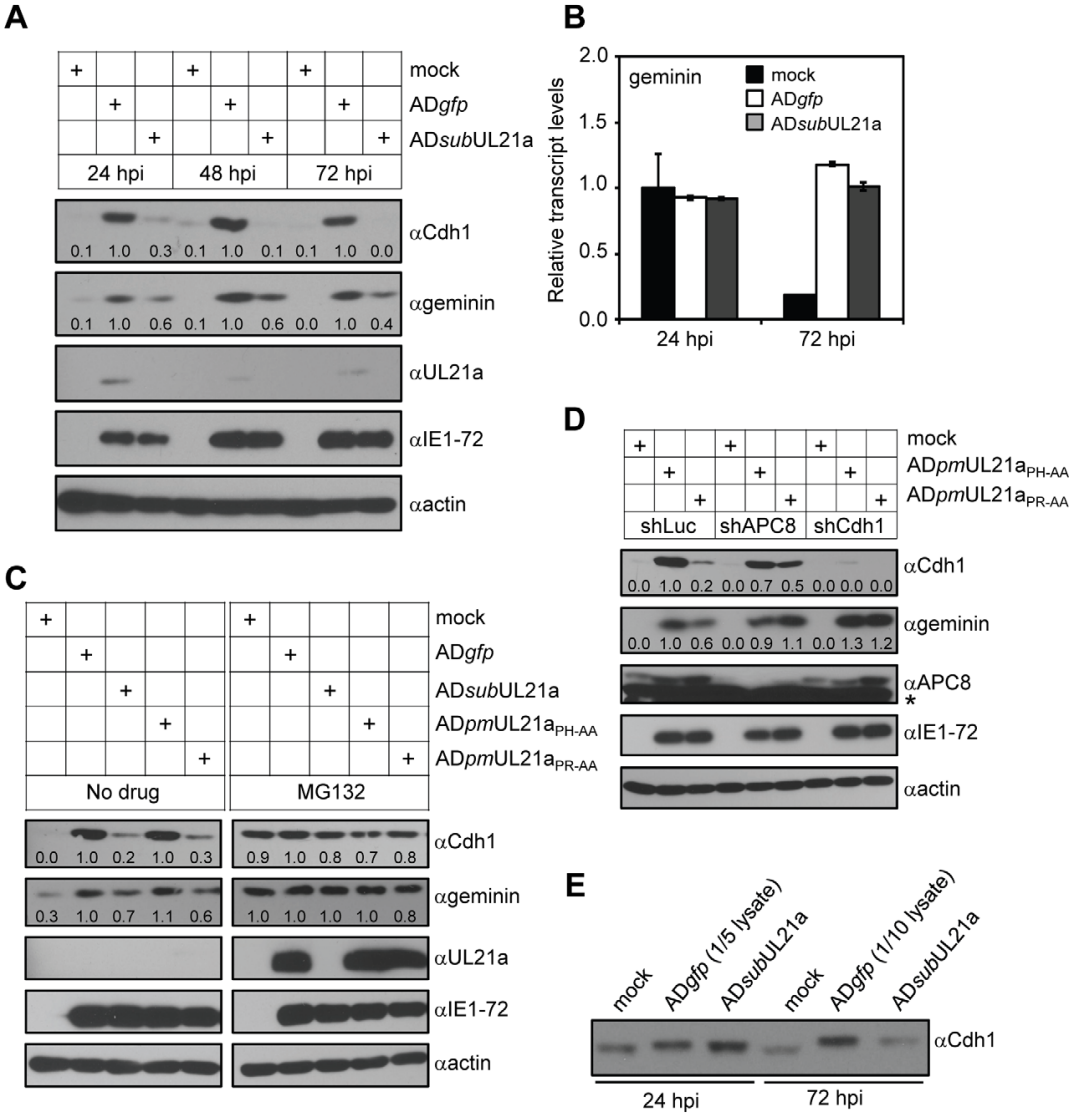
pUL21a regulates APC activity during HCMV infection. (A) pUL21a is required for elevated accumulation of APC substrate proteins in HCMV infection. MRC-5 cells were infected with AD*gfp* or AD*sub*UL21a, and cell lysates were collected at indicated times and analyzed by immunoblotting. Protein bands were quantified using Image J software and normalized to the wild-type value at each time point. Results were reproducible in four independent experiments. (B) pUL21a is not required for geminin transcript accumulation. MRC-5 cells were infected with AD*gfp* or AD*sub*UL21a, and total RNA was collected at indicated times. Geminin transcript was measured by RT-qPCR and normalized to that of GAPDH. (C) Proteasome-dependent degradation of APC substrates is dependent on the APC binding activity of pUL21a. MRC-5 cells were infected with AD*gfp*, AD*sub*UL21a, AD*pm*UL21a_PH-AA_, or AD*pm*UL21a_PR-AA_ in the presence or absence of MG132. Cell lysates were collected at 20 hpi and analyzed by immunoblotting. Protein bands were quantified using Image J software and normalized to the wild-type value in each condition. Results were reproducible in three independent experiments. (D) APC knockdown restores APC substrate accumulation in UL21a mutant virus infection. MRC-5 cells were transduced with lentivirus expressing the indicated shRNA (see Materials and Methods for shRNA sequence). After 48 hours, cells were infected with AD*pm*UL21a_PH-AA_ or AD*pm*UL21a_PR-AA_, and cell lysates were collected at 72 hpi and analyzed by immunoblotting. Protein bands were quantified as in panel A with values normalized to that of shLuc-expressing cells infected with AD*pm*UL21a_PH-AA._ Results were reproducible in two independent experiments. (E) Immunoblot analysis of Cdh1 from infected cells. One-fifth or one-tenth equivalent of lysate from AD*gfp*-infected cells relative to that from AD*sub*UL21a-infected cells at 24 or 72 hpi, respectively, was loaded on the SDS-PAGE to differentiate the migration patterns of Cdh1. For all of the quantitative analyses, the representative results from at least two independent experiments are shown.

To determine the functional consequence of pUL21a-dependent APC dissociation, we first analyzed the accumulation of APC substrates during wild-type or UL21a-deletion virus infection. The protein levels of APC substrates Cdh1 (that is also an APC co-activator) and geminin were markedly increased in wild-type virus infection as previously reported [27], [33] (Figure 4A). However, their levels were reduced during infection with the UL21a-deletion virus, suggesting increased APC activity. The geminin transcript accumulated to wild-type levels even without pUL21a, providing evidence that the difference in protein accumulation was not due to transcriptional regulation (Figure 4B). PAA treatment had no effect on substrate accumulation, ruling out pUL21a-mediated late gene expression as the source of the observed phenotype (Figure S5A). MG132 largely restored substrate levels during UL21a deletion viral infection, indicating that the difference is likely due to increased proteasome degradation (Figure 4C). These results were also recapitulated during infection of APC binding mutant virus AD*pm*UL21a_PR-AA_ and its control virus AD*pm*UL21a_PH-AA_ (Figure 4C).

To confirm that decreased APC substrate accumulation during mutant virus infection was due to APC activity, we used shRNAs to knock down APC8 or the coactivator Cdh1 to deplete APC activity. Both APC8 and Cdh1 shRNAs efficiently reduced expression of their respective targets (Figures 4D and S5B). Importantly, APC8 knockdown restored geminin and Cdh1 levels in cells infected with AD*pm*UL21a_PR-AA_ or AD*sub*UL21a virus to those with AD*pm*UL21a_PH-AA_ or AD*gfp* virus. Likewise, Cdh1 knockdown restored geminin levels in cells infected with the pUL21a-deficient viruses. Thus, our results indicate that pUL21a association with the APC allows it to target APC4 and APC5 subunits for degradation to alter APC activity during infection.

It is noteworthy that not all APC substrates were subjected to pUL21a-mediated regulation. We did not observe significant difference in Cdc6 or a drastic reduction in thymidine kinase protein levels in the UL21a mutant relative to wild-type viral infection (data not shown). It is possible that these APC substrates are regulated by multiple mechanisms, including APC-independent viral regulation, pUL21a-mediated alteration in APC substrate specificity, and pUL97-mediated phosphorylation of the APC coactivator Cdh1. In fact, Cdh1 from both wild type and UL21a mutant virus infected cells migrated slower in an SDS-PAGE gel compared to that from mock cells, which was previously shown to be due to phosphorylation (Figure 4E) [28]. Therefore, virus-induced, Cdh1 phosphorylation-mediated APC regulation appears intact even without pUL21a during HCMV infection.

As the APC prevents the premature entry of the cell cycle into S phase, we predicted that increased APC activity in the absence of pUL21a would not compromise the ability of HCMV to arrest infected cells at G1/S phase boundary. Consistent with this hypothesis, cells infected with wild type, AD*pm*UL21a_PH-AA_, or AD*pm*UL21a_PR-AA_virus showed indistinguishable cell cycle profiles throughout infection, with the majority of cells phenotypically arrested in G1 phase (Figure S6).

### pUL21a Is Sufficient to Reduce APC4 and APC5 Protein Levels and Alter APC Activity

To test if pUL21a was sufficient to alter APC activity, we first analyzed 293T cells that over-expressed pUL21a by transient transfection. Expression of pUL21a alone was sufficient to markedly reduce the levels of APC4 and APC5 (Figure S7A), and as expected, geminin and Cdh1 levels were elevated in these cells. These pUL21a-expressing cells were largely arrested in G2/M phase (Figure S7B), failed to multiply, and ultimately died (Figure S7C). The biological characteristics of pUL21a-expressing cells are therefore consistent with reduced APC activity.

**Figure 5:**
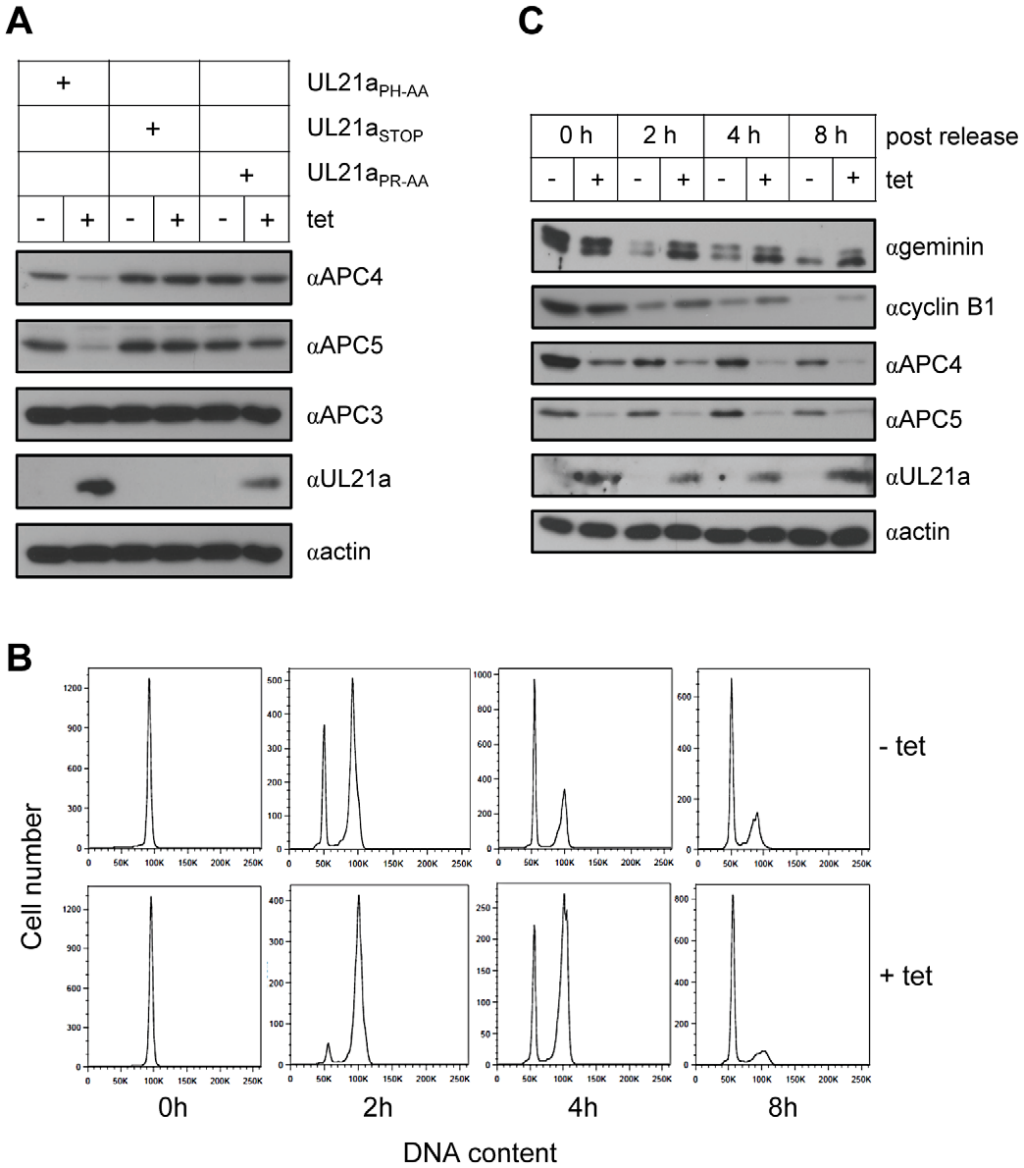
pUL21a reduces APC4 and APC5 protein levels and inhibits APC activity in an inducible cell line. (A) pUL21a expression is sufficient to reduce APC4 and 5 protein accumulation. Hela cells constitutively expressing GFP-tagged TetR were transduced with pLKO-derived lentivirus that expressed pUL21a_PH-AA_, pUL21a_stop_, or pUL21a_PR-AA_ under a Tet operator (TetO)-regulated CMV promoter. Transduced cells were enriched by antibiotic selection and pUL21a expression was induced by tetracycline treatment for 72 hours. Cell lysates were collected and analyzed by immunoblotting. (B) pUL21a expression is sufficient to induce an M-phase cell cycle arrest. Cells carrying pUL21a_PH-AA_ (+/− tetracycline) were treated with nocodazole (100 ng/µl) for 16 hours to synchronize cells in G2/M phase. The cells were then released from nocodazole, collected at indicated time points, and analyzed for DNA content by flow cytometry. (C) pUL21a expression is sufficient to regulate APC activity. Cells from panel B were also analyzed for APC subunit and substrate levels by immunoblotting.

To more precisely test if pUL21a was able to regulate the APC in the absence of other HCMV proteins, we developed an inducible pUL21a expression system. We constructed a HeLa cell line stably expressing a GFP-tagged TetR (tetracycline repressor) gene. We then transduced this cell line with lentiviruses expressing pUL21a_stop_, pUL21a_PH-AA_, or pUL21a_PR-AA_ under a CMV-TetO (tetracycline operator) promoter. pUL21a protein accumulation was only detected in the presence of tetracycline, suggesting tight regulation of pUL21a expression (Figure 5A), although its levels were significantly lower than those expressed in transiently transfected cells (Figure S7A). Importantly, the addition of tetracycline significantly reduced APC4 and APC5 protein levels in cells expressing pUL21a_PH-AA_, but not pUL21a_stop_ or pUL21a_PR-AA_. To assess the consequence of pUL21a on APC activity, we synchronized cells expressing pUL21a_PH-AA_ (i.e. wild-type pUL21a) in mitosis with nocodazole and then assayed their ability to progress out of mitosis after release from nocodazole treatment. In the absence of tetracycline and pUL21a, cells readily progressed through the mitotic phase following release. In the experiment shown in Figure 5B, 26% and 48% of cells entered the next G1 phase by 2 and 4 hours, respectively. In the presence of tetracycline where pUL21a was expressed, progression through the mitotic phase was clearly delayed. As the result, only 5% and 24% of cells reached G1 by 2 and 4 hours, even though by 8 hours most of pUL21a-expressing cells were able to enter G1, likely due to low expression of pUL21a in these cells as compared to those in transiently transfected cells. Additionally, following nocodazole withdrawal, APC substrates geminin and cyclin B1 remained elevated in the presence of tetracycline while their levels were reduced in its absence (Figure 5C). Our results provide strong evidence that pUL21a expression alone is sufficient to regulate APC activity.

### Abrogation of Both pUL21a APC Regulatory Activity and pUL97 Results in a More Severe Attenuation in HCMV Growth than pUL97 Deletion Alone

**Figure 6:**
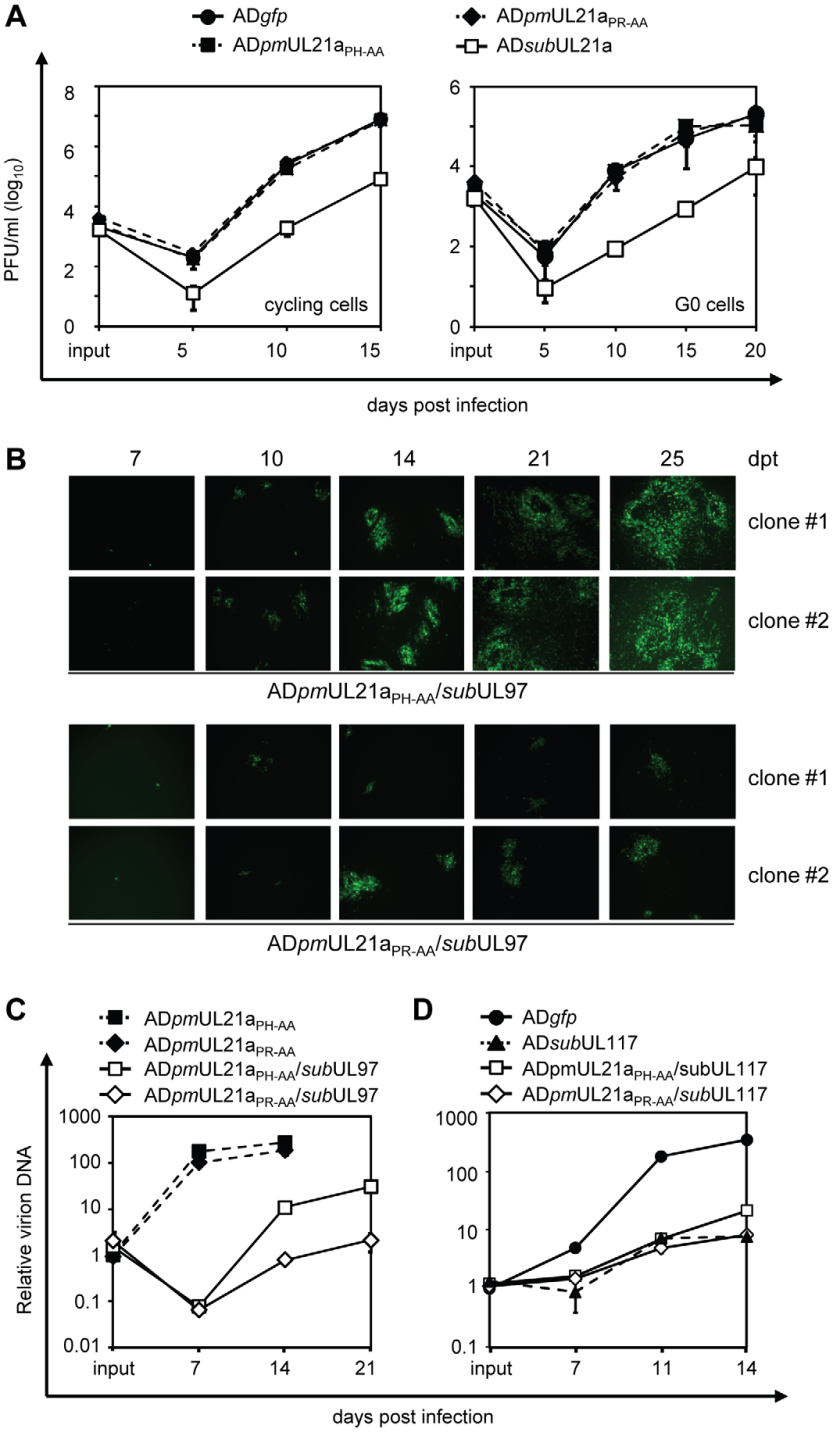
Abrogation of both pUL21a APC regulatory activity and pUL97 results in a more severe attenuation in HCMV growth than pUL97 deletion alone. (A) Abrogation of pUL21a-APC binding alone is not sufficient to alter HCMV replication. MRC-5 cells in serum-containing (cycling condition) or serum-free (G0 condition) media were infected with AD*gfp*, AD*sub*UL21a, AD*pm*UL21a_PH-AA_, or AD*pm*UL21a_PR-AA_ at an MOI of 0.01. Production of cell-free virus at indicated times was determined by plaque assay. (B) Abrogation of both UL97 and the pUL21a-APC binding site markedly reduced the efficiency of HCMV reconstitution as compared to abrogation of UL97 alone. To reconstitute AD*pm*UL21a_PR-AA_/*sub*UL97 and pAD*pm*UL21a_PH-AA_/*sub*UL97 viruses, MRC-5 fibroblasts were transfected with their corresponding BAC clones. For each recombinant virus, three independent clones were tested. Shown are representative images of virus spread indicated by virus-driven GFP expression at indicated days post transfection of two of the three clones. Images were taken under a Leica fluorescent microscope. (C) Abrogation of both UL97 and the pUL21a-APC binding site markedly reduced HCMV replication as compared to abrogation of UL97 alone. MRC-5 cells were infected with indicated recombinant viruses at an input genome number equivalent to that of 0.03 infectious units of wild type virus/cell. Production of cell-free virion DNA at indicated times was determined by qPCR analysis and normalized to input levels of AD*pm*UL21a_PH-AA_, which was set to 1. (D) Multi-step growth analysis of double mutant viruses that carried the UL117 deletion and point mutation in the UL21a-APC binding site. Cells were infected with indicated recombinant viruses and analyzed as described in panel C. The input value of AD*gfp* was set to 1.

In the final experiments, we tested the consequence of pUL21a-mediated APC regulation on HCMV replication in fibroblasts. We first tested if the ability of pUL21a to regulate the APC would be responsible for its previously reported role in promoting viral DNA replication [30]. We compared the growth of AD*pm*UL21a_PR-AA_ mutant virus (i.e. pUL21a APC-binding deficient) to that of wild-type, AD*pm*UL21a_PH-AA_ (i.e. pUL21a APC-binding competent), or UL21a deletion viruses in multi-step growth curve analysis. We found that AD*pm*UL21a_PR-AA_ grew indistinguishably from wild-type and AD*pm*UL21a_PH-AA_ viruses in both cycling and G0-synchronized fibroblasts, whereas the UL21a deletion virus had a 100-fold defect (Figure 6A) [29]. Furthermore, knockdown of Cdh1 and APC8 was unable to enhance UL21a-deletion virus replication (data not shown). This suggests that pUL21a has at least two independent activities. One is to facilitate viral DNA replication via an unknown mechanism and is responsible for the growth defect of UL21a deletion virus. The second activity is to regulate the APC, whose impact on virus replication is not apparent under the aforementioned experimental conditions.

**Figure 7:**
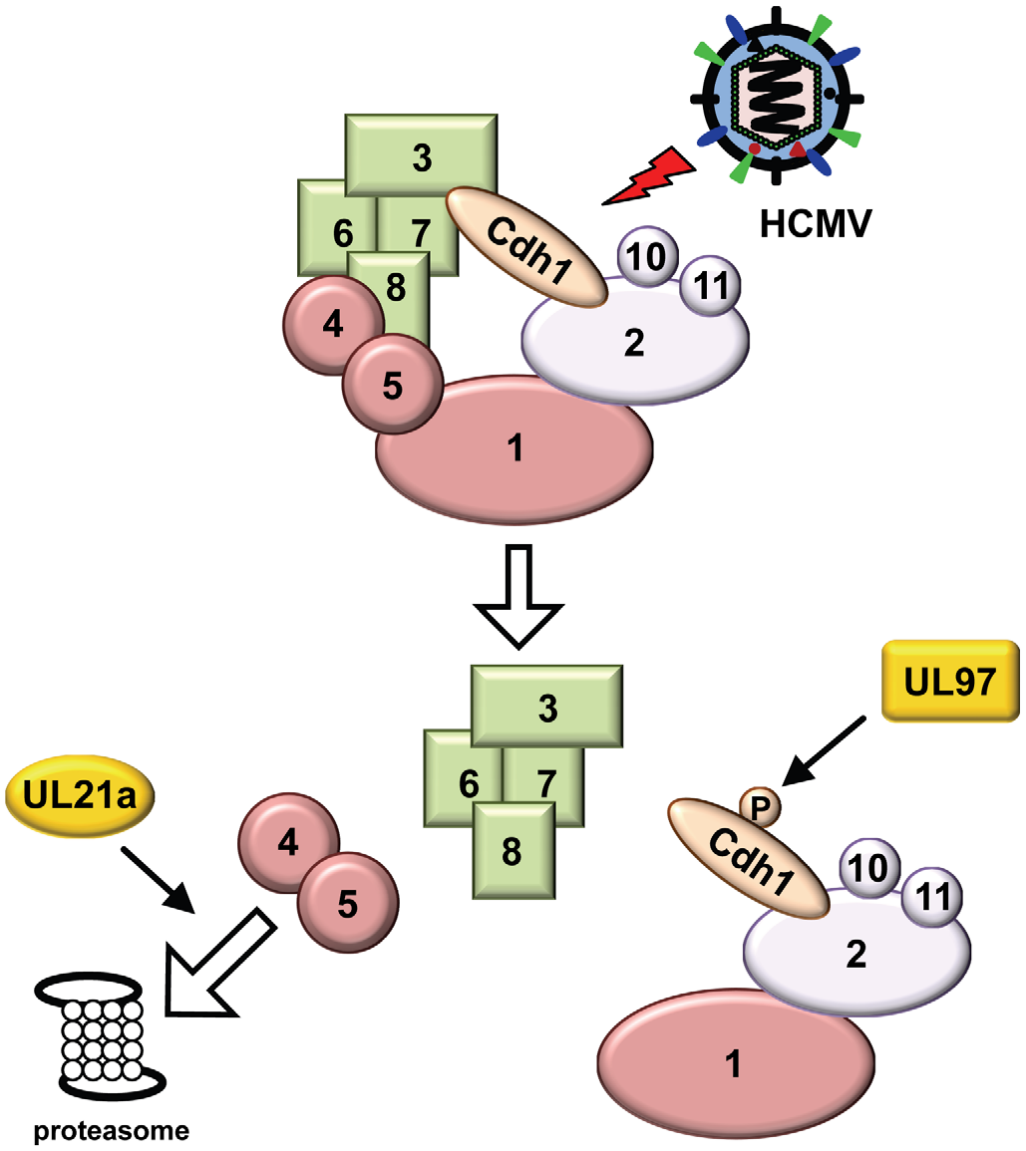
Working model of virus-mediated APC regulation during HCMV infection. HCMV uses two mechanisms to regulate the APC during infection. The pUL97 viral kinase inhibits the APC co-activator Cdh1 by phosphorylation while pUL21a targets APC4 and APC5 for proteasome-dependent degradation to dissociate the complex.

As two HCMV proteins, pUL97 and pUL21a, are capable of regulating the APC, we hypothesized that one of these two proteins acted to compensate for the loss of the other during infection. Consistent with this hypothesis, HCMV appeared to retain the ability, at least to some extent, to regulate the APC even when pUL21a or pUL97 is absent (Figure 4E, and data not shown) [24]. To test this hypothesis more directly, we created recombinant HCMV viruses AD*pm*UL21a_PH-AA_/*sub*UL97 and AD*pm*UL21a_PR-AA_/*sub*UL97. The two viruses were derived from AD*pm*UL21a_PH-AA_ and AD*pm*UL21a_PR-AA_, respectively, and both contained an additional deletion in UL97. Both recombinant viruses grew slower than wild-type virus due to lack of the multifunctional pUL97 protein (Figure 6C). However, reconstitution of AD*pm*UL21a_PR-AA_/*sub*UL97 that lacked pUL21a APC-binding activity following BAC transfection was markedly slower than that of AD*pm*UL21a_PH-AA_/*sub*UL97 (Figure 6B). At day 25 post transfection, while cells transfected with the BAC clone of AD*pm*UL21a_PH-AA_/*sub*UL97 showed nearly 100% of CPE indicated by virus-driven GFP expression, GFP-positive foci in cells transfected with the BAC clone of AD*pm*UL21a_PR-AA_/*sub*UL97 were distinctly smaller. Furthermore, multi-step growth curve analysis showed that titers of AD*pm*UL21a_PR-AA_/*sub*UL97 were 13- and 14- fold lower than that of AD*pm*UL21a_PH-AA_/*sub*UL97 at 14 and 21 days post infection (dpi), respectively (Figure 6C). As a control to show that this phenotype was not due to general viral attenuation resulting from the UL97 deletion, we also constructed double mutant viruses AD*pm*UL21a_PH-AA_/*sub*UL117 and AD*pm*UL21a_PR-AA_/*sub*UL117. These two viruses were derived similarly from AD*pm*UL21a_PH-AA_ and AD*pm*UL21a_PR-AA_, but also contained a deletion in viral gene UL117. We chose UL117 as the control because its mutation attenuated virus growth but not viral early or early-late gene expression so UL97 expression was unlikely affected [34]. BAC transfection reconstituted both mutant viruses at similar efficiency and produced viruses with similar titers (data not shown). Multi-step growth analysis demonstrated that AD*pm*UL21a_PH-AA_/*sub*UL117 and AD*pm*UL21a_PR-AA_/*sub*UL117 replicated at similar kinetics (Figure 6D). At 14 dpi, the titer of AD*pm*UL21a_PR-AA_/*sub*UL117 was slightly lower (e.g. 1.5-fold) than that of AD*pm*UL21a_PH-AA_/*sub*UL117. However, growth of mutant virus carrying only the UL117 deletion tracked with AD*pm*UL21a_PR-AA_/*sub*UL117, suggesting that the difference between the PH and PR mutants at 14 dpi, if any, is minimal. Together, our data provide evidence that disruptions of both pUL97 and the APC regulatory activity of pUL21a are synthetically lethal to HCMV replication. This is consistent with a working model that these two functions enable HCMV to cope with APC activity to promote virus replication (Figure 7).

In sum, we have shown that the HCMV protein pUL21a antagonizes the APC by promoting proteasome-mediated disruption of this prominent cellular E3 ubiquitin ligase.

## Discussion

HCMV has been shown to have two different means to regulate the anaphase-promoting complex (APC) during infection [24], [27], [28]. It can induce phosphorylation of APC co-activator Cdh1, and it induces dissociation of the complex by promoting proteasomal degradation of two components of the bridge subcomplex, APC4 and APC5. The viral protein pUL97 appears to be responsible for Cdh1 phosphorylation [24]. However, pUL97 is an HCMV-encoded kinase that has many reported roles [26], [35]. How this particular pUL97 activity impacts HCMV infection remains elusive. Importantly, the viral factor or precise molecular mechanism mediating APC4 and APC5 degradation has not been identified, and how APC disruption contributes to HCMV replication is not known. Here, we have identified the HCMV protein pUL21a as the viral factor that mediates APC disruption. It does so by interacting with the APC and inducing proteasome-dependent degradation of APC4 and APC5, which results in complex dissociation. This is the first identified viral protein that modulates the APC in this manner. We also show, for the first time, the impact of viral modulation of the APC, particularly by pUL21a, on HCMV replication. Loss of pUL21a-mediated APC regulation has minimal impact on virus replication but the combined loss of both pUL97- and pUL21a-mediated regulation markedly attenuates growth of the virus relative to single loss of pUL21a- or pUL97- mediated regulation. Our studies support a working model in which HCMV uses pUL97-mediated Cdh1 phosphorylation and pUL21a-mediated complex disruption to control APC activity for efficient virus infection (Figure 7). Why has HCMV developed these two distinct mechanisms that seemingly lead to a similar biological consequence? It is possible that these two mechanisms have differential roles in HCMV infection under different conditions or in particular cell types, even though either one seems sufficient and can compensate for loss of the other in fibroblasts. Alternatively, it is possible that these two mechanisms serve as the fallback for one another or act synergistically to maximize the ability of the virus to acquire a complete control of the APC during infection. In any event, the fact that HCMV uses multiple means to subvert the APC underlines its critical role in HCMV infection. This is particularly true for large DNA viruses such as HCMV, which often encode multiple viral factors to regulate the same or related cellular targets central to their infection [36]. However, it is often challenging to dissect these intertwined viral mechanisms during infection because of the presence of other factors targeting the same process. The regulation of the APC represents one such critical but complex viral regulatory strategy, and our studies shed light into its role and mechanism during HCMV infection.

Several viral factors from different viral families have been reported to use diverse mechanisms to regulate the APC. For instance, the human papillomavirus E2 protein binds to and inhibits the Cdh1 activator protein [20], while the parapoxvirus virus protein PACR (poxviral APC regulator) functions as an enzymatically inactive APC11 mimic [23], [37]. The chicken anemia virus (CAV) protein apoptin can bind to the APC at the bridge and cause its dissociation using an unknown mechanism [19]. The fact that proteins from both HCMV and CAV target the APC bridge subcomplex suggests that viruses have evolved regulatory strategies converging on this sub-complex as an efficient means to disable APC activity. It is intriguing to speculate that modulating the APC complex by dissolving the bridge may allow viruses to alter substrate specificity of the APC instead of completely abolishing its activity, as the enzymatic portion of APC is known to have activity *in vitro* [23], [38]. HCMV does not appear to directly destroy the enzymatic subcomplex of APC, so it is of interest to determine if the APC retains some activity or is directed to target different substrates during virus infection.

Several viral proteins have now been reported to regulate the APC in overexpression, and evidence correlating the role of these factors and viral replication is emerging. Deletion of the parapoxvirus PACR or CAV protein apoptin markedly attenuated virus growth in tissue culture even though their ability and role in inhibiting the APC during infection has not been clarified [23], [39]. Recently, the UL97 kinase of HCMV has been shown to phosphorylate Cdh1 and partially inhibit the APC during infection but with unknown consequences for viral replication [24]. Our study elucidates the mechanism by which pUL21a regulates APC in the context of virus infection and indicates a role of this pUL21a activity in viral replication. Mutation abolishing the APC binding activity of pUL21a had no impact on viral growth in tissue culture, but the loss of both pUL21a-APC binding and pUL97 markedly attenuated viral replication relative to the loss of pUL97 alone. Our data suggest that HCMV has evolved a sophisticated strategy by encoding both pUL97 and pUL21a to overcome APC activity. However, further experiments are needed to unequivocally demonstrate the vital role of APC regulation in HCMV replication and provide mechanistic insight into how this regulation impacts its biology.

How does pUL21a target APC4 and APC5 for proteasome degradation? pUL21a does not contain a sequence domain that would suggest it as an E3 ligase, thus likely ruling out this possibility. Currently, we also do not know which subunit of the APC complex that pUL21a directly binds to so the precise mechanism that it uses to degrade APC4 and APC5 remains elusive. It is certainly possible that pUL21a may bind to a subunit neighboring to APC4 and APC5 so it can disrupt the APC structure leading to APC4 and APC5 degradation, or recruit a protein degradation enzyme (e.g. E3 ubiquitin ligase) to destabilize the subunits. However, knockdown of APC8 does not abrogate the ability of pUL21a to degrade APC4 and APC5, suggesting that APC8 is not involved and the presence of the entire complex is not required. Intriguingly, pUL21a itself is a highly unstable protein and likely degraded in a ubiquitin-independent manner [29], [40]. It is tempting to speculate that pUL21a may directly bind APC4 and APC5 and target them for degradation in a ubiquitin-independent manner. One focus of future work is to identify the APC component that pUL21a directly binds to and elucidate the mechanism of how pUL21a targets APC4 and APC5 to the proteasome.

What would be the benefit for the virus to alter APC activity? The APC may restrict HCMV replication via several mechanisms. The APC not only promotes cell cycle progression through M phase, it also prevents cells from prematurely entering S phase. Thus virus-mediated APC regulation may help HCMV maintain an S phase-like cellular environment for viral replication. The APC targets more than 40 proteins for degradation, so it may deplete host factors critical to viral replication. Consequently, viruses may need to alter the substrate specificity of the APC or allow accumulation of APC substrates critical for viral replication. Interestingly, the only viruses within the poxvirus and herpesvirus families that are known to modulate the APC (e.g. parapoxviruses and HCMV) are those that do not encode viral thymidine kinase (TK) and ribonucleotide reductase subunit M2 (RRM2). Both enzymes are APC substrates and critical for the production of deoxyribonucleotides. It is tempting to speculate that this viral regulation of the APC may provide viruses a means to produce sufficient nucleotides to replicate their genome [23], [27]. Nonetheless, the APC also targets proteins involved in cellular DNA synthesis, glycolysis and glutaminolysis, and cell cycle progression, all of which could impact viral replication [41]. Moreover, the APC may also promote ubiquitination and degradation of viral proteins to restrict infection [42]. Several HCMV proteins contain a putative destruction Box (D-box) motif, an APC recognition signal commonly found in its substrates [24]. Future work is needed to differentiate these possibilities and unravel the APC substrates that may be critical for viral replication.

Insight into the mechanism of pUL21a-mediated APC regulation may also have broad impact on cancer and neuronal disease. Due to its essential role in cell cycle progression, the APC is a promising target for novel anti-cancer therapeutics [16], [43]. In fact, we found in this study that overexpression of pUL21a essentially prevented the proliferation of a transformed cell line (Figure S7), suggesting that pUL21a regulation of the APC could inhibit cancer cell growth. Furthermore, several recent studies have also highlighted a vital role of the APC in neuronal development (for a review, see [44]). HCMV infects neuronal cells and congenital HCMV infection leads to neuronal disease and severe complications such as blindness, hearing loss, and mental retardation. It is reasonable to speculate that inhibition of the APC by pUL21a may play a role in promoting neuronal disease in congenitally infected infants. Therefore, an understanding of pUL21a-APC interaction may reveal novel mechanisms of APC assembly and regulation, give further impetus to target the APC for anti-cancer therapies, and uncover new insights into the molecular basis of HCMV pathogenesis.

## Materials and Methods

### Plasmids and Reagents

Primary embryonic lung fibroblasts (MRC-5), human newborn foreskin fibroblasts (HFFs), 293T, and Hela cells were propagated in Dulbecco's modified Eagle medium (DMEM) supplemented with 10% fetal bovine serum, non-essential amino acids, and penicillin-streptomycin. Transient transfection of expression constructs were carried out using lipofectamine according to the manufacturers' instructions.

pYD-C235 is a pLPCX-derived retroviral vector (Clontech) that expresses a DsRed gene driven by an internal ribosome entry site 2 (IRES2) [45]. pYD-C474 was created by PCR amplifying the coding sequence of the pGFP-UL21a fusion protein from pAD*gfp*UL21a (see below) and ligating it into the multiple cloning site of pYD-C235. pYD-C580 was created by replacing the coding sequence of wild-type UL21a in pYD-C474 with that of mutant UL21a carrying two stop-codon mutations at the N-terminus (i.e. UL21a_stop_) [30]. Vectors expressing pGFP-UL21a truncation mutants were derived from pYD-C235 while vectors expressing point mutants were derived from pYD-C474. Truncation mutants were made by PCR amplifying the targeted UL21a coding sequences and point mutants were created using a QuickChange XL kit (Stratagene). Primers used to create these mutants are listed in Table S2. pYD-C160, pYD-C175, and pYD-C682 are pRetro-EBNA derived retroviral expression vectors that expressed GFP, UL21a, and UL21a_stop_, respectively. pYD-C648 and pYD-C649 are pLKO-based lentiviral vectors expressing GFP-TetR and carrying the CMV-TetO_2_ promoter, respectively (generous gifts from Roger Everett, University of Glasgow Centre for Viral Research) [46]. YD-C665, YD-C667, and YD-C669 are lentiviral expression vectors created by cloning the UL21a_stop_, UL21a_PH-AA_, and UL21a_PR-AA_ sequences into the multiple cloning site of YD-C649. To produce pLKO-based lentiviruses, 293T cells were transfected with corresponding pLKO vectors along with packaging plasmids. Lentivirus was collected at 48 and 72 hours and used to transduce MRC-5 cells. To create GFP-TetR expressing stable cells, Hela cells were transduced with pYD-C648 derived lentivirus and sorted for GFP expression 48 hours later. GFP-positive cells were collected, grown in the presence of G418 (500 µg/ml), and frozen as cells stably expressing GFP-TetR. These stable cells were then transduced with lentivirus derived from YD-C665, YD-C667, and YD-C669, selected with puromycin (2 µg/ml), and tested for tetracycline (1 µg/ml)-regulated expression of targeted genes.

For shRNA knockdown, MRC-5 cells were transduced with pLKO-based lentivirus expressing shRNA against the targeted gene for 48 hours. The shRNA sequence for Cdh1 knockdown was 5′CCAGTCAGAACCGGAAAGCCA3′ and the shRNA sequence for APC8 knockdown was 5′GCAGGAGGTAATATGCTATAA3′. All pLKO-based shRNA lentiviral vectors were purchased from the Washington University Children's Discovery Institute/Genome Center.

The primary antibodies used in this study included anti-β actin (AC-15, Abcam); anti-HA (HA.11, Covance); anti-GFP (3E6 and A6455, Invitrogen); anti-APC3 (AF3.1, Santa Cruz and 610454, BD); anti-APC8 (6114, Biolegend); anti-APC4 (A301-176A, Bethyl laboratories); anti-APC5 (A301-026A, Bethyl laboratories); anti-geminin (sc-13015, Santa Cruz); anti-Cdh1 (DH01, Calbiochem); anti-cyclin B1 (ms868 P1, Thermo-Scientific); anti-UL21a [29]; anti-IE2 (mAB8140, Chemicon); and anti-IE1 and anti-pp28 (generous gifts from Thomas Shenk, Princeton University) [45]. Phosphonoacetic acid (PAA), MG132, tetracycline, gancyclovir (GCV), and propidium iodide (PI) were purchased from Sigma-Aldrich. Lipofectamine 2000 and Protein A-conjugated Dynabeads were purchased from Invitrogen.

### Recombinant HCMV Viruses

Recombinant HCMV AD169 viruses were reconstituted from transfection of corresponding BAC-HCMV clones as previously described [34]. Viral stocks were prepared by ultra-centrifugation of infected culture supernatant through 20% D-sorbitol cushion and re-suspending pelleted virus in serum-free medium. The following BAC-HCMV clones were used in the present study, and were constructed using PCR-based linear recombination as previously reported [29], unless indicated otherwise. pAD-GFP, which carried the GFP-tagged genome of the HCMV AD169 strain, was used to produce wild-type virus AD*gfp* [45]. pAD*gfp*UL21a, which carried an N-terminally GFP-tagged version of pUL21a, was used to produce AD*gfp*UL21a virus [29]. pAD*sub*UL21a, which carried a GalK/kanamycin dual mutagenic cassette in place of the UL21a coding sequence, was used to produce UL21a-deletion virus AD*sub*UL21a [29]. pAD*gfp*UL21a_PR-AA_, pAD*gfp*UL21a_PH-AA_, pAD*pm*UL21a_PR-AA_, or pAD*pm*UL21a_PH-AA_ carried point mutation PR109-110AA or PH111-112AA in the GFP tagged or native UL21a gene, respectively. These recombinant BAC clones were used to produce corresponding point mutant viruses. pAD*pm*UL21a_PH-AA_/*sub*UL97 and pAD*pm*UL21a_PR-AA_/*sub*UL97 carried the GalK/kanamycin mutagenic cassette in place of UL97 on the background of pAD*pm*UL21a_PR-AA_ and pAD*pm*UL21a_PH-AA_ BAC clones. Similarly, pAD*pm*UL21a_PH-AA_/*sub*UL117, pAD*pm*UL21a_PR-AA_/*sub*UL117, and pAD*sub*UL117 carried the GalK/kanamycin mutagenic cassette in place of UL117 on the background of pAD*pm*UL21a_PR-AA_, pAD*pm*UL21a_PH-AA_, and pAD-GFP BAC clones, respectively. All BACs were confirmed by restriction digestion, PCR, and sequencing. HCMV virus titers were determined in duplicate in HFFs by tissue culture infectious dose 50 (TCID_50_) assay or plaque assay. Relative viral genome numbers were determined by real-time quantitative PCR (qPCR) as described previously [29].

### HCMV Infection

For most infections, subconfluent MRC-5 cells in serum-containing medium were inoculated with recombinant HCMV virus at an input genome number equivalent to that of 3–5 infectious units of wild type virus/cell for 1 hour, unless otherwise indicated. Inoculum was removed and fresh medium was replenished. For infection of G0-synchronized cells, MRC-5 cells were incubated in serum-free medium for 72 hours, infected as described above, and maintained in serum-free media throughout the infection. For shRNA knockdown experiments, subconfluent MRC-5 cells were transduced with lentivirus for 24 hours, incubated in fresh medium for additional 48 hours, and infected as described above. When necessary, PAA (100 µg/ml) was added immediately following infection, and MG132 (10 µM) was added 12–14 hours prior to harvest. For viral growth analysis, virus production in the media of infected cultures was determined by TCID_50_, plaque assay, or qPCR. For qPCR analysis, virion DNA was prepared as previously described [29]. Briefly, cell-free supernatants were treated with DNase I to remove contaminating DNA, and virions were lysed with proteinase K and SDS. DNA was extracted with phenol/chloroform/isoamyl alcohol and precipitated with ethanol. The DNA was subjected to qPCR using primers and a taqman probe specific for UL54.

### Protein Analysis

For immunoprecipitation, frozen cell pellets were lysed in lysis buffer (0.5% NP-40, 50 mM Tris-Cl pH 8.0, 125 mM NaCl, supplemented with protease and phosphatase inhibitors) using an end-over-end rotator at 4°C for 30 minutes. Cell extracts were cleared by centrifugation at 16,000× g for 15 minutes. Supernatants were incubated with protein A-coated Dynabeads that were coupled to 1 µg anti-HA (HA.11, Covance), 1 µg anti-GFP (3E6, Invitrogen) or 2 µg anti-APC3 (AF3.1, Santa Cruz) mouse monoclonal antibodies at 4°C for 1–2 hours. Beads were washed with PBS and immunoprecipitated protein complexes were eluted by boiling beads in reducing sample buffer for 5 minutes. Cell extracts (pre-IP) were also collected and boiled in reducing sample buffer. For mass spectrometry analysis, protein complexes were resolved by SDS-polyacrylamide gel electrophoresis (Invitrogen) followed by staining with a silver stain kit (Sigma-Aldrich). Protein bands specific to immunoprecipitated pUL21a complex were excised for identification by MS/MS mass spectrometry [47].

For immunoblotting, total cell or pre-IP extracts were lysed in sample buffer containing SDS and protease and phosphatase inhibitors. Proteins were resolved on a SDS polyacrylamide gel, transferred to a polyvinylidene difluoride (PVDF) membrane, hybridized with a primary antibody, reacted with the horseradish peroxidase-conjugated secondary antibody, and visualized using chemiluminescent substrate (Thermo Scientific).

### Reverse Transcription Coupled-Quantitative PCR Analysis (RT-qPCR)

Total RNA was extracted with TRIzol (Invitrogen) and treated with Turbo DNA-free reagent (Ambion) to remove genomic DNA contaminants. cDNA was reverse transcribed from total RNA with random hexamer primers using the High Capacity cDNA reverse transcription kit (Applied Biosystems). cDNA was quantified using SYBR Advantage qPCR Premix (Clontech) and primers for the cellular genes geminin, APC4, APC5, and GAPDH (glyceraldehyde-3-phosphate dehydrogenase) as an internal control (see below). cDNA from infected cells was used to generate a standard curve for each gene examined. The standard curve was then used to calculate the relative amount of specific RNA present in a sample.

Primers used for RT-qPCR are as follows: geminin, forward 5′GCCTTCTGCATCTGGATCTCTT3′ and reverse 5′CGATGTTTCCTTTTGGACAAGC3′ [24]; APC4, forward 5′ATTCTCGTCCTTGGAGGAAGCTCT3′ and reverse 5′TTCTGGCCATCCGAGTTACTTCAG3′ [24]; APC5, forward 5′GTGCCATGTTCTTAGTGGCCAAGT3′ and reverse 5′GATGCGCTCTTTGCAGTCAACCTT-3′ [24]; GAPDH, forward 5′CTGTTGCTGTAGCCAAATTCGT3′ and reverse 5′ACCCACTCCTCCACCTTTGAC3′ [30].

### Analysis of Cellular DNA Content

To determine cellular DNA content, cells were trypsinized, collected by low-speed centrifugation, fixed, and permeabilized in ice-cold 70% ethanol overnight. Cells were stained with propidium iodide only, or double-stained with propidium iodide and anti-pUL44 antibody to identify HCMV-infected cells. Total or pUL44-positive cells were determined for their DNA content by cell-cycle analysis with flow-cytometry. Percentages of cells in each cell cycle compartment were calculated using CellQuest or FlowJo software.

## Supporting Information

Figure S1 APC8 knockdown by shRNA. MRC-5 cells were transduced with lentivirus expressing shRNA targeting either Luc (negative control) or APC8. Forty-eight hours post transduction, cells were infected with mock, wild-type (AD*gfp*), or UL21a-deletion virus (AD*sub*UL21a). Cell lysates were collected at 72 hpi and analyzed by immunoblotting. Note that the asterisk-marked bottom band that reacted with the APC8 antibody was nonspecific as it was not affected by the APC8-targting shRNA.
(TIF)

Figure S2 Amino acid residues PR_109–110_ of pUL21a are required for its APC binding during HCMV infection. Cells were infected with indicated virus and MG132 was added to the final concentration of 10 µM at 6 hpi. Cells were collected at 20 hpi and lysates were immunoprecipitated with APC3 antibody. Cell lysates and eluted proteins were analyzed by immunoblotting.
(TIF)

Figure S3 pUL21a dissociates the APC by promoting degradation of the bridge subcomplex. MRC-5 cells were infected with AD*gfp* or AD*sub*UL21a, and MG132 was added to the final concentration of 10 µM at 6 hpi. Cells were collected at 20 hpi and lysates were immunoprecipitated with APC3 antibody. Both cell lysates and eluted proteins were analyzed by immunoblotting.
(TIF)

Figure S4 APC8 is not required for pUL21a-mediated degradation of APC4 and APC5. Knockdown and subsequent immunoblot were performed as described in the legend to Figure S1.
(TIF)

Figure S5 pUL21a regulates APC activity during HCMV infection. (A) Reduced accumulation of APC substrates during UL21a mutant virus infection is not due to a defect in viral late gene expression. MRC-5 cells were infected with AD*gfp* or AD*sub*UL21a in the presence or absence of PAA. Cells were collected at 72 hpi, and lysates were analyzed by immunoblotting. (B) APC knockdown restores APC substrate accumulation during UL21a mutant virus infection. MRC-5 cells were transduced with lentivirus expressing indicated shRNA. 48 hours post transduction, cells were infected with AD*gfp* or AD*sub*UL21a. Cells were collected at 72 hpi, and lysates were analyzed by immunoblotting. Protein bands were quantified using Image J software and normalized to the value of shLuc-expressing cells infected with AD*gf*p virus. Results were reproducible in three independent experiments.
(TIF)

Figure S6 Loss of pUL21a-mediated APC regulation does not compromise HCMV's ability to block cellular DNA synthesis. (A) Cell cycle profiles at 48 hpi of MRC-5 cells that were mock infected or infected with AD*gfp*, AD*pm*UL21a_PH-AA_, or AD*pm*UL21a_PR-AA_. (B) Percentage of cells in each compartment of the cell cycle at 24 and 48 hpi.
(TIF)

Figure S7 Transient expression of pUL21a reduces APC4 and APC5 protein levels and inhibits cell proliferation. 293T cells were transfected with plasmid expressing GFP, UL21a, or UL21a_stop_, and selected with puromycin treatment for 72 hours. (A) Analysis of indicated protein accumulation by immunoblotting. (B) Analysis of cellular DNA content by flow cytometry. (C) Analysis of cell proliferation by plating 1×10^5^ cells and counting cells at indicated days.
(TIF)

Table S1 pUL21a interacting proteins identified by mass spectrometry.
(DOC)

Table S2 Primers used to create mutations in UL21a.
(DOC)
