# Supplementary figures and images for: Proteasome-Dependent Disruption of the E3 Ubiquitin Ligase Anaphase-Promoting Complex by HCMV Protein pUL21a
Source: PLoS Pathog. 2012 Jul 5;8(7):e1002789. doi: 10.1371/journal.ppat.1002789 (PMC3390409; doi:10.1371/journal.ppat.1002789)

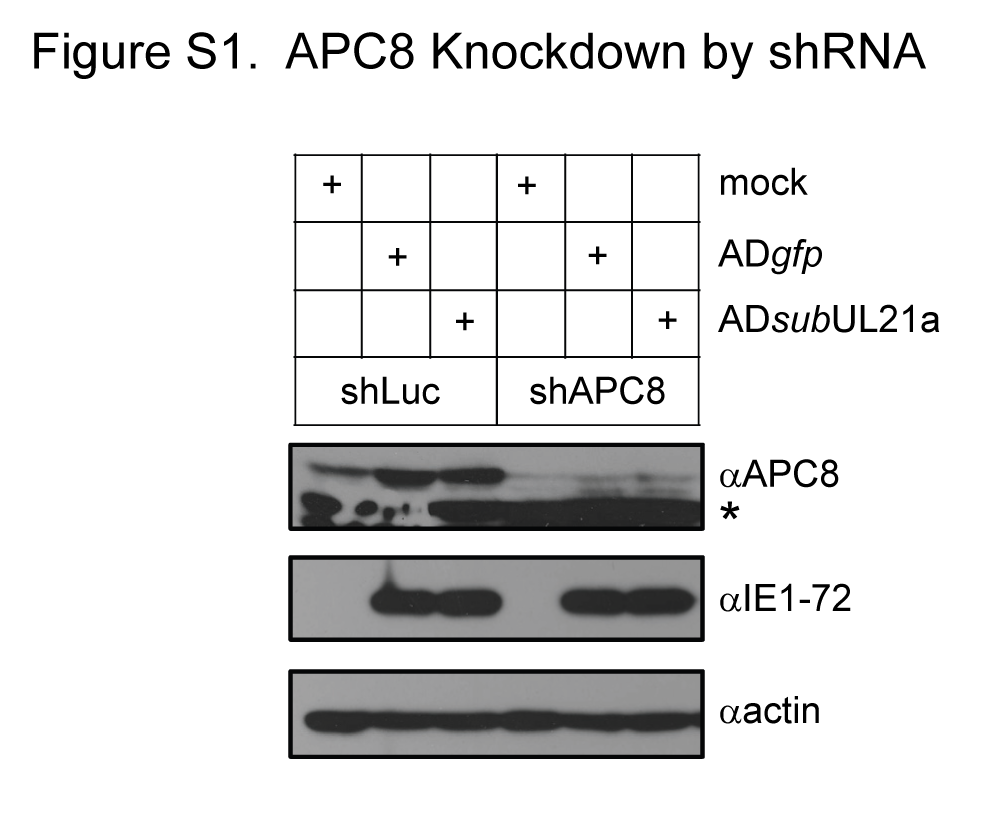

Supplement: Figure S1 — APC8 knockdown by shRNA. MRC-5 cells were transduced with lentivirus expressing shRNA targeting either Luc (negative control) or APC8. Forty-eight hours post transduction, cells were infected with mock, wild-type (ADgfp), or UL21a-deletion virus (ADsubUL21a). Cell lysates were collected at 72 hpi and analyzed by immunoblotting. Note that the asterisk-marked bottom band that reacted with the APC8 antibody was nonspecific as it was not affected by the APC8-targting shRNA. (TIF) [file ppat.1002789.s001.tif]

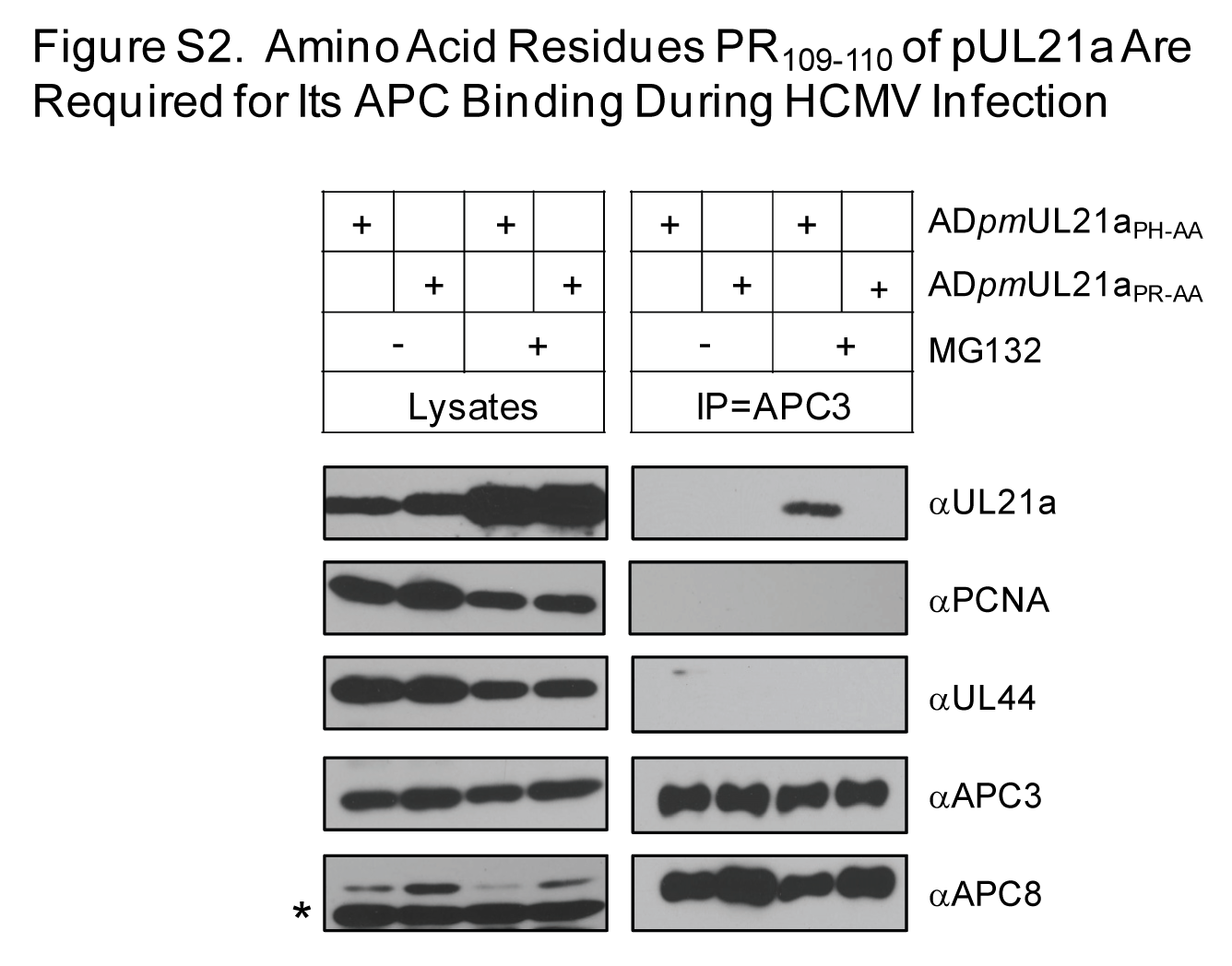

Supplement: Figure S2 — Amino acid residues PR109–110 of pUL21a are required for its APC binding during HCMV infection. Cells were infected with indicated virus and MG132 was added to the final concentration of 10 µM at 6 hpi. Cells were collected at 20 hpi and lysates were immunoprecipitated with APC3 antibody. Cell lysates and eluted proteins were analyzed by immunoblotting. (TIF) [file ppat.1002789.s002.tif]

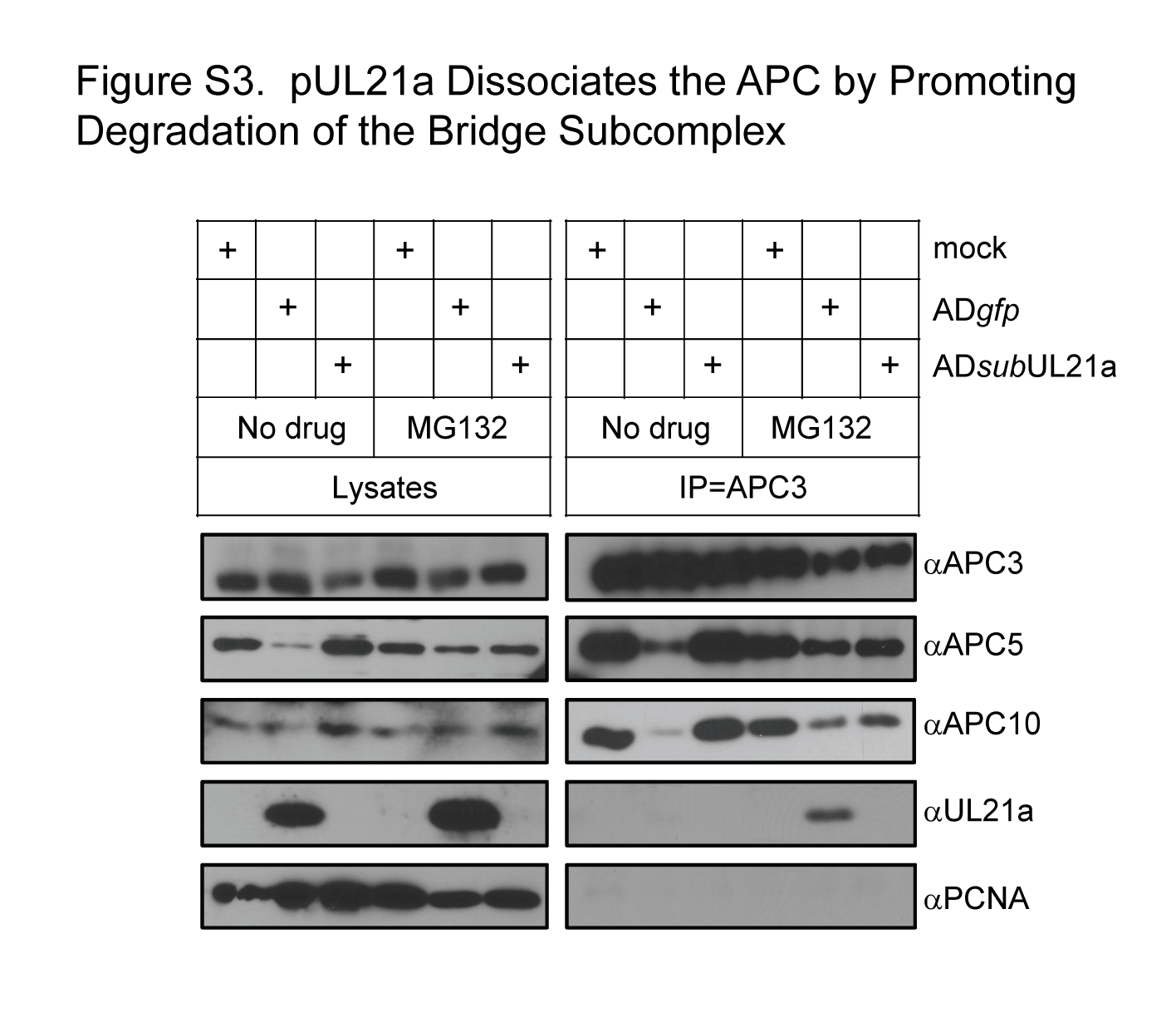

Supplement: Figure S3 — pUL21a dissociates the APC by promoting degradation of the bridge subcomplex. MRC-5 cells were infected with ADgfp or ADsubUL21a, and MG132 was added to the final concentration of 10 µM at 6 hpi. Cells were collected at 20 hpi and lysates were immunoprecipitated with APC3 antibody. Both cell lysates and eluted proteins were analyzed by immunoblotting. (TIF) [file ppat.1002789.s003.tif]

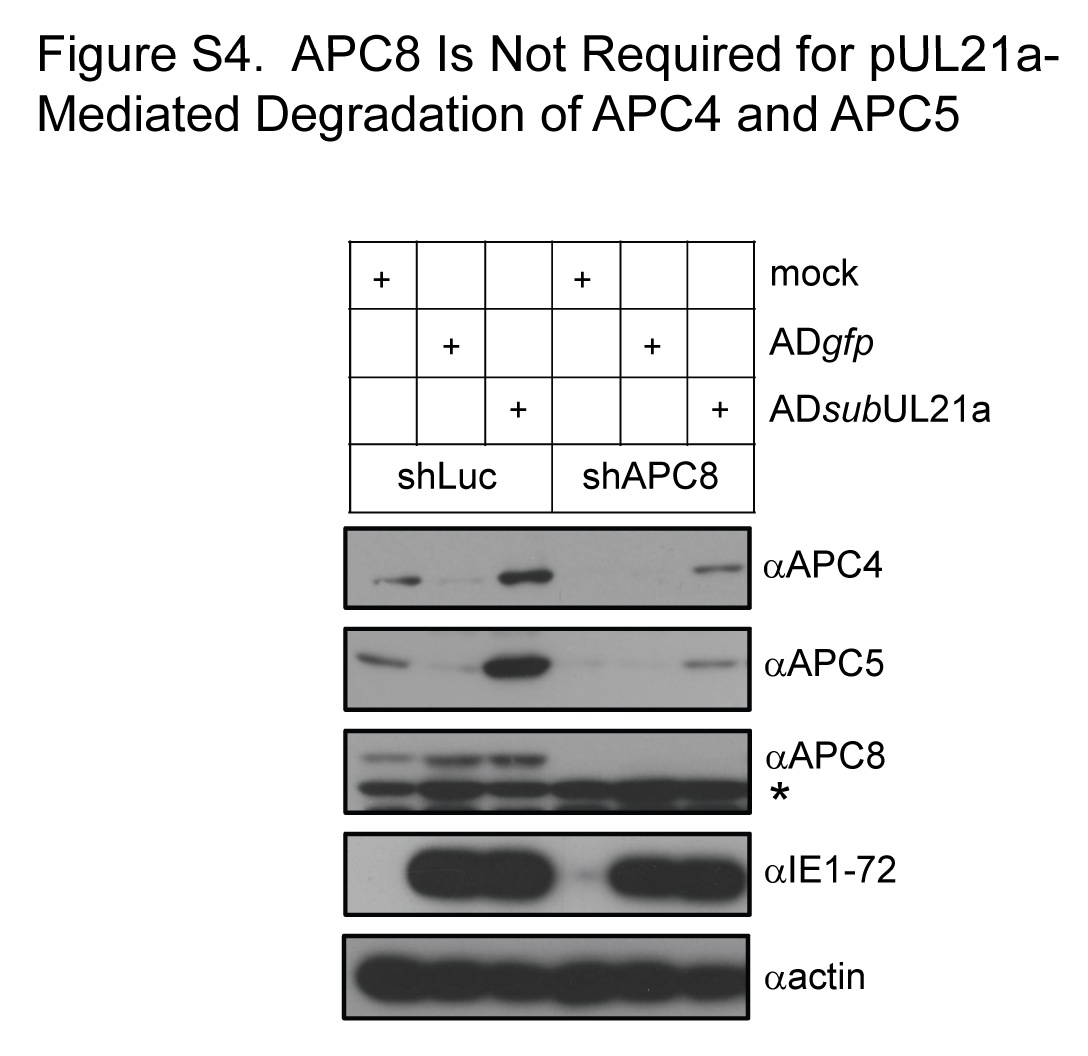

Supplement: Figure S4 — APC8 is not required for pUL21a-mediated degradation of APC4 and APC5. Knockdown and subsequent immunoblot were performed as described in the legend to Figure S1. (TIF) [file ppat.1002789.s004.tif]

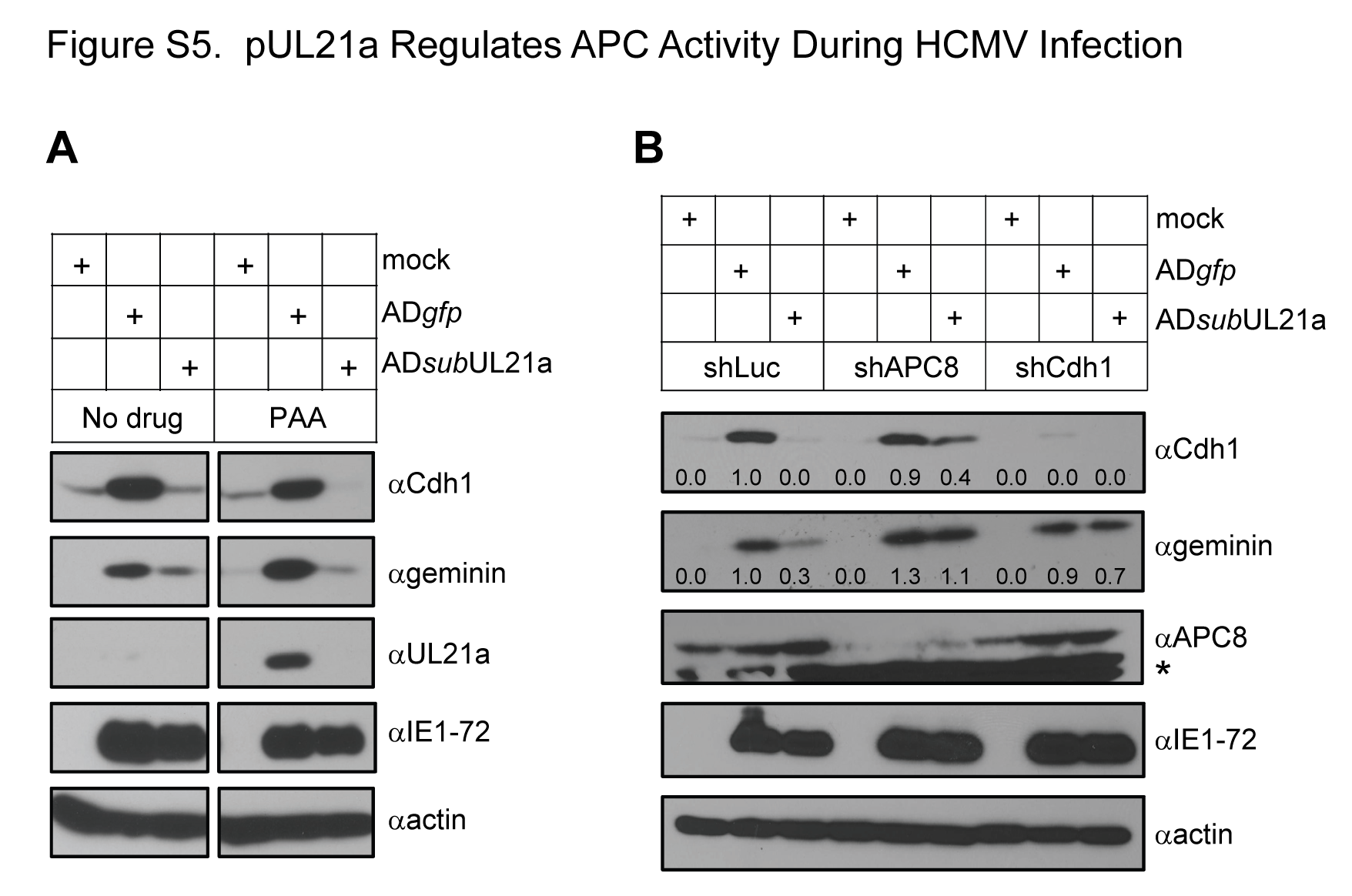

Supplement: Figure S5 — pUL21a regulates APC activity during HCMV infection. (A) Reduced accumulation of APC substrates during UL21a mutant virus infection is not due to a defect in viral late gene expression. MRC-5 cells were infected with ADgfp or ADsubUL21a in the presence or absence of PAA. Cells were collected at 72 hpi, and lysates were analyzed by immunoblotting. (B) APC knockdown restores APC substrate accumulation during UL21a mutant virus infection. MRC-5 cells were transduced with lentivirus expressing indicated shRNA. 48 hours post transduction, cells were infected with ADgfp or ADsubUL21a. Cells were collected at 72 hpi, and lysates were analyzed by immunoblotting. Protein bands were quantified using Image J software and normalized to the value of shLuc-expressing cells infected with ADgfp virus. Results were reproducible in three independent experiments. (TIF) [file ppat.1002789.s005.tif]

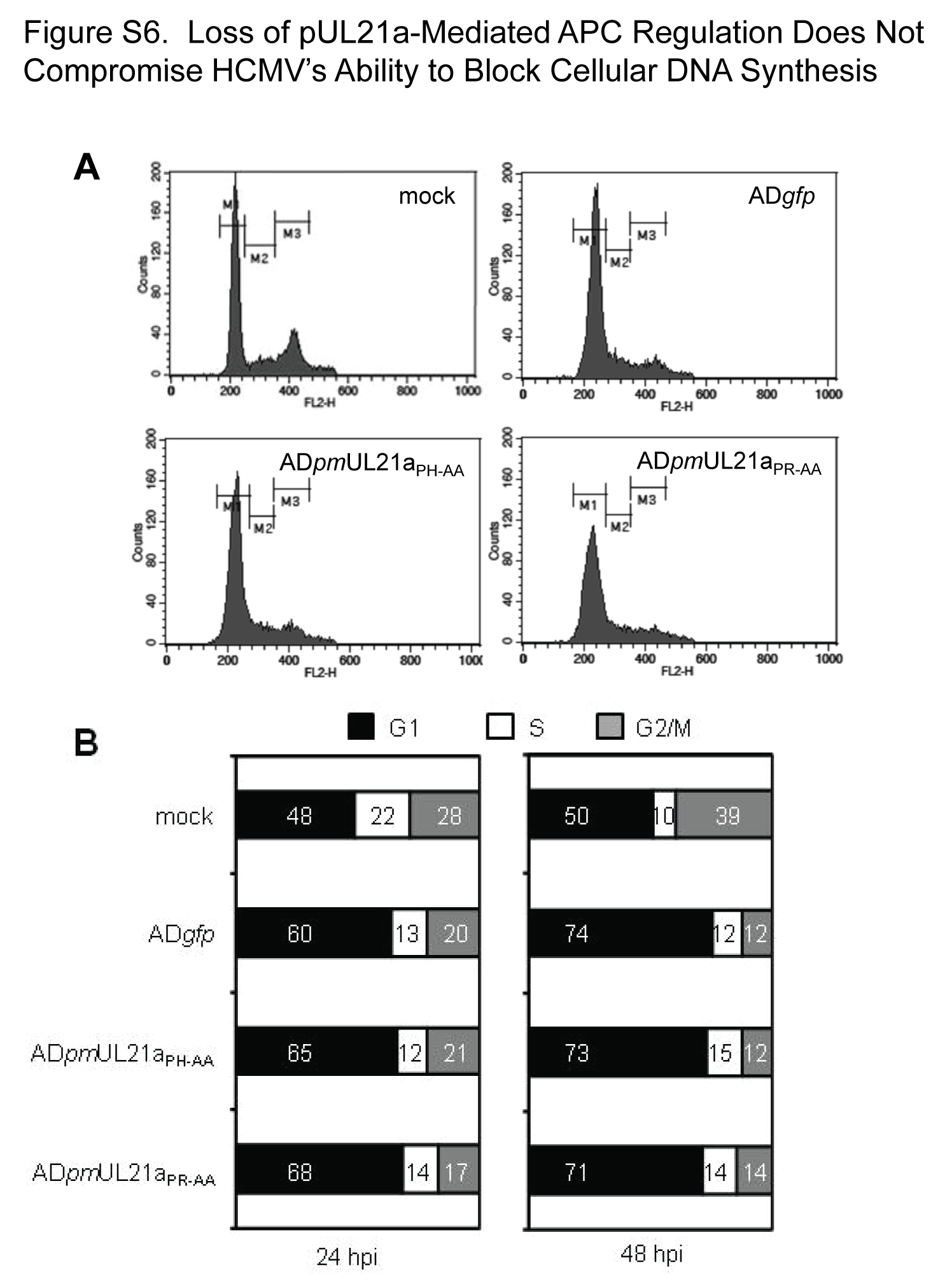

Supplement: Figure S6 — Loss of pUL21a-mediated APC regulation does not compromise HCMV's ability to block cellular DNA synthesis. (A) Cell cycle profiles at 48 hpi of MRC-5 cells that were mock infected or infected with ADgfp, ADpmUL21aPH-AA, or ADpmUL21aPR-AA. (B) Percentage of cells in each compartment of the cell cycle at 24 and 48 hpi. (TIF) [file ppat.1002789.s006.tif]

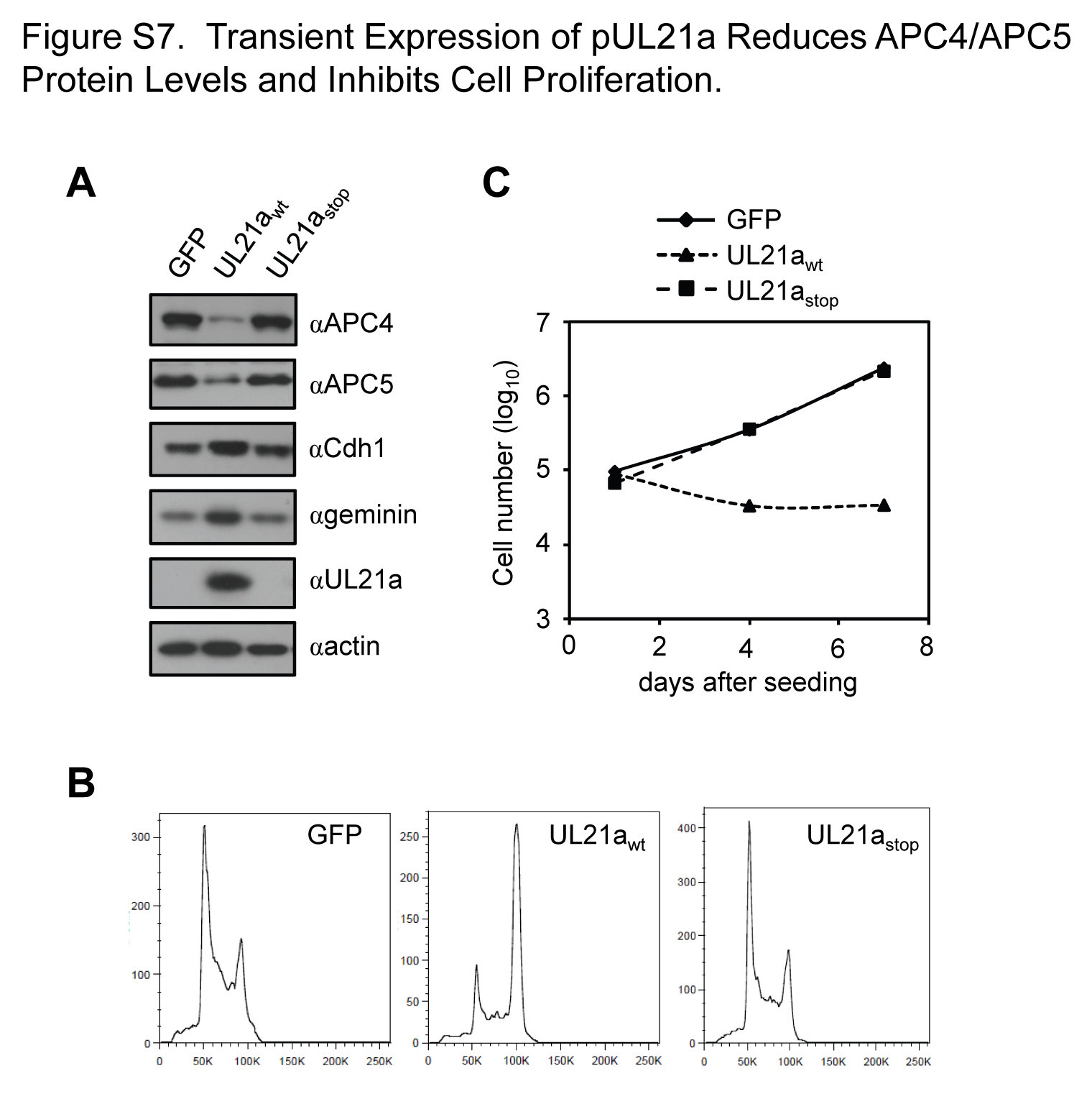

Supplement: Figure S7 — Transient expression of pUL21a reduces APC4 and APC5 protein levels and inhibits cell proliferation. 293T cells were transfected with plasmid expressing GFP, UL21a, or UL21astop, and selected with puromycin treatment for 72 hours. (A) Analysis of indicated protein accumulation by immunoblotting. (B) Analysis of cellular DNA content by flow cytometry. (C) Analysis of cell proliferation by plating 1×10^5^ cells and counting cells at indicated days. (TIF) [file ppat.1002789.s007.tif]
