# Supplementary material for: Proteasome-Dependent Disruption of the E3 Ubiquitin Ligase Anaphase-Promoting Complex by HCMV Protein pUL21a
Source: PLoS Pathog. 2012 Jul 5;8(7):e1002789. doi: 10.1371/journal.ppat.1002789 (PMC3390409; doi:10.1371/journal.ppat.1002789)
Supplement: Table S1 — pUL21a interacting proteins identified by mass spectrometry. (DOC) [file ppat.1002789.s008.doc]

**Table S1. pUL21a interacting proteins identified by mass spectrometry.**

| Name  (Accession) | Full Name  (Alternative symbols) | Peptides | Peptide Sequence | Scans | Coverage | Molecular Weight (Da) |
| --- | --- | --- | --- | --- | --- | --- |
| CDC27  (P30260) | Cell division cycle 27 homolog (APC3; HNUC; NUC2; ANAPC3; CDC27Hs; D0S1430E; D17S978E) | 6 | ISTITPQIQAFNLQK  LAEGEQILSGGVFNK  SALQELEELK  GGITQPNINDSLEITK  DAVFLAER  DVALSVLSK | 1  1  1  1  1  1 | 8.86 | 91810.34 |
| CDC23  (Q9UJX2) | Cell division cycle 23 homolog (APC8, CUT23, ANAPC8) | 4 | QLLLIAGLTR  IENMDTFSNLLYVR  NQGETPTTEVPAPFFLPASLSANNTPTR  LHEQLTESEQAAQCYIK | 2  1  1  1 | 11.68 | 68242.77 |
| ANAPC7  (Q9UJX3) | Anaphase promoting complex subunit 7 (APC7) | 2 | AIQLNSNSVQALLLK  VRPSTGNSASTPQSQCLPSEIEVK | 1  1 | 6.90 | 63093.84 |
