# Supplementary material for: Proteasome-Dependent Disruption of the E3 Ubiquitin Ligase Anaphase-Promoting Complex by HCMV Protein pUL21a
Source: PLoS Pathog. 2012 Jul 5;8(7):e1002789. doi: 10.1371/journal.ppat.1002789 (PMC3390409; doi:10.1371/journal.ppat.1002789)
Supplement: Table S2 — Primers used to create mutations in UL21a. (DOC) [file ppat.1002789.s009.doc]

**Table S2. Primers used to create mutations in UL21a**.

| Mutation type | Primer pairs to introduce mutations *^a^* | Construct name |
| --- | --- | --- |
| Truncation; expressing the N-terminal fragment of 1-47 aa | 5’gtgactcgagATGGGAGGTAGCCCTGTTCC3’  5’ggaattcttaATTTTGGAAAAGCCTCCGAC3’ | pLP-UL21a_N(1-47)_ |
| Truncation; expressing the middle region fragment of 46-87 aa | 5’gtgactcgagCAAAATCATATACATCCAGAA3’  5’ggaattctta ATTGGGTAGGGGTTGCTGCGG3’ | pLP-UL21a_M(46-87)_ |
| Truncation; expressing the C-terminal fragment of 84-123aa | 5’gtgactcgagCCCCTACCCAATCCGCTGGTG3’  5’ggaattcTTAAAACTGGTCCCAATGTTCTT3’ | pLP-UL21a_C(84-123)_ |
| Truncation; expressing the fragment of 1-87 aa | 5’gtgactcgagATGGGAGGTAGCCCTGTTCC3’  5’ggaattcTTAATTGGGTAGGGGTTGCTGCGG3’ | pLP-UL21a_NM(1-87)_ |
| Point mutation; PL84-85AA | 5’CGACCTCCGCAGCAAGCCGCACCCAATCCGCTGGTG3’  5’CACCAGCGGATTGGGTGCGGCTTGCTGCGGAGGTCG3’ | pLP-UL21a_PL-AA_ |
| Point mutation; PLV88-90AAA | 5’GCAACCCCTACCCAATGCGGCGGCGCTACTGCTGGACGATG3’  5’CATCGTCCAGCAGTAGCGCCGCCGCATTGGGTAGGGGTTGC3’ | pLP-UL21a_PLV-AAA_ |
| Point mutation; LLL91-93AAA | 5’CTACCCAATCCGCTGGTGGCAGCGGCGGACGATGTTCCCCCCCAT3’  5’ATGGGGGGGAACATCGTCCGCCGCTGCCACCAGCGGATTGGGTAG3’ | pLP-UL21a_LLL-AAA_ |
| Point mutation; DD94-95AA | 5’CTGGTGCTACTGCTGGCCGCTGTTCCCCCCCATGTA3’  5’TACATGGGGGGGAACAGCGGCCAGCAGTAGCACCAG3’ | pLP-UL21a_DD-AA_ |
| Point mutation; PR109-110AA | 5’GCTCCTTACCGCGTCGCCGCTCCCCACCCCATGATT3’  5’AATCATGGGGTGGGGAGCGGCGACGCGGTAAGGAGC3’ | pLP-UL21a_PR-AA_ |
| Point mutation; PH111-112AA | 5’TACCGCGTCCCCCGTGCCGCCCCCATGATTCCCGAA3’  5’TTCGGGAATCATGGGGGCGGCACGGGGGACGCGGTA3’ | pLP-UL21a_PH-AA_ |

*^a^* Restriction sites are in lowercase, UL21a specific sequences are in upper case, and base pair changes to introduce mutations are underlined and in upper case.
